# Radical-mediated C-S bond cleavage in C2 sulfonate degradation by anaerobic bacteria

**DOI:** 10.1038/s41467-019-09618-8

**Published:** 2019-04-08

**Authors:** Meining Xing, Yifeng Wei, Yan Zhou, Jun Zhang, Lianyun Lin, Yiling Hu, Gaoqun Hua, Ankanahalli N. Nanjaraj Urs, Dazhi Liu, Feifei Wang, Cuixia Guo, Yang Tong, Mengya Li, Yanhong Liu, Ee Lui Ang, Huimin Zhao, Zhiguang Yuchi, Yan Zhang

**Affiliations:** 1 Tianjin Key Laboratory for Modern Drug Delivery & High-Efficiency, Collaborative Innovation Center of Chemical Science and Engineering, School of Pharmaceutical Science and Technology, Tianjin University, 300072 Tianjin, China; 2 Metabolic Engineering Research Laboratory, Institute of Chemical and Engineering Sciences, Agency for Science, Technology and Research (A*STAR), Singapore, 138669 Singapore; 3 Technical Institute of Physics and Chemistry, Chinese Academy of Sciences, Beijing, 100190 China; 4 Department of Chemical and Biomolecular Engineering, University of Illinois at Urbana-Champaign, 600 South Mathews Avenue, Urbana, IL 61801 USA

## Abstract

Bacterial degradation of organosulfonates plays an important role in sulfur recycling, and has been extensively studied. However, this process in anaerobic bacteria especially gut bacteria is little known despite of its potential significant impact on human health with the production of toxic H_2_S. Here, we describe the structural and biochemical characterization of an oxygen-sensitive enzyme that catalyzes the radical-mediated C-S bond cleavage of isethionate to form sulfite and acetaldehyde. We demonstrate its involvement in pathways that enables C2 sulfonates to be used as terminal electron acceptors for anaerobic respiration in sulfate- and sulfite-reducing bacteria. Furthermore, it plays a key role in converting bile salt-derived taurine into H_2_S in the disease-associated gut bacterium *Bilophila wadsworthia*. The enzymes and transporters in these anaerobic pathways expand our understanding of microbial sulfur metabolism, and help deciphering the complex web of microbial pathways involved in the transformation of sulfur compounds in the gut.

## Abstract (web summary)

The C2 sulfonates taurine and isethionate are also present in the anaerobic mammalian gut, where they are converted into toxic H_2_S by sulfate and sulfite-reducing bacteria. Here the authors characterise the O_2_-sensitive enzyme IseG that catalyzes the C-S bond cleavage of isethionate and show that IseG also plays a key role in converting taurine into H_2_S in *Bilophila wadsworthia*.

## Introduction

The C2 sulfonates aminoethylsulfonate (taurine) and hydroxyethylsulfonate (isethionate) are widespread in the environment, originating from both biotic and industrial sources. Despite the chemical inertness of the sulfonate C–S bond, microbes have evolved a variety of enzymes and pathways to incorporate the carbon and sulfur into their metabolism, and play an important role in the degradation of these C2 sulfonates^1,2^. Isethionate, as a fatty acyl ester, is a component of common industrial surfactants, and is present in some habitats as an industrial pollutant^2^, where bacterial metabolism accelerates mineralization of the sulfonate sulfur, returning it to the sulfur cycle.

Taurine and isethionate are also present in the human body, and have great relevance to human health. Taurine is a key osmolyte in mammals, and one of the most abundant amino acids in the human body^3^. Excretion of taurine takes place through the gut in the form of taurine-conjugated bile salts^4^, stimulated by a diet high in meat and fat. Hydrolysis by bacterial bile salt hydrolases releases taurine as a major constituent of the sulfur pool available to gut bacteria. Isethionate in the human body is thought to be a product of taurine metabolism by gut bacteria^5^. While carbon and nitrogen are largely fermented to innocuous end-products, the sulfonate sulfur are converted by the consortium of anaerobic gut bacteria into toxic H_2_S^4^ implicated in inflammation, colorectal cancer and gastrointestinal dysbiosis^4,6^.

The rich enzymology of bacterial taurine and isethionate degradation has been studied over the past five decades^7^, revealing diverse strategies for C–S bond cleavage (Supplementary Fig. 1). O_2_-dependent enzymes such as TauD and SsuD^8,9^ catalyze C–S cleavage of taurine and isethionate respectively, through activated oxygen species generated under aerobic conditions (Supplementary Fig. 1a). Sulfoacetaldehyde acetyltransferase (Xsc)^10^ catalyzes C–S cleavage of sulfoacetaldehyde, a common intermediate in both taurine and isethionate degradation, to form sulfite and acetyl-phosphate (Supplementary Fig. 1b, c). The catalytic mechanism of Xsc requires a thiamine pyrophosphate cofactor, and occurs under both aerobic and anaerobic conditions.

Despite its importance, the microbial transformation of C2 sulfonate sulfur in anoxic environments, which includes a significant fraction of the biosphere and the human gut, remains poorly understood. Sulfonates are particularly important substrates for sulfate- and sulfite-reducing bacteria (SSRB)^1,2^, which are strict anaerobes that use sulfite as a terminal electron acceptor (TEA) for anaerobic respiration, reducing it to H_2_S. In certain environments, such as the gut environment and marine microbial mats^11^, sulfonates constitute a major source of oxidized sulfur compounds for SSRB metabolism.

Two decades ago, it was found that certain SSRB could use taurine and isethionate as TEAs, reducing the sulfonate sulfur to H_2_S^1,2^. Preliminary experiments suggested that the pathway requires sulfonate C–S cleavage, liberating sulfite for subsequent reduction by the dissimilatory sulfite reductase^12^. However, the complete pathways for anaerobic taurine and isethionate metabolism in SSRB have not yet been determined. In particular, many SSRB do not possess Xsc, necessitating new enzymes that catalyze C–S cleavage without relying on O_2_.

In this work, we report the identification of an O_2_-sensitive isethionate sulfo-lyase, which catalyzes C–S cleavage of isethionate via a radical-dependent mechanism. We further demonstrate its involvement in pathways for the degradation of isethionate and taurine by prominent SSRB from the human gut.

## Results

### Identification of GUF as a candidate sulfonate C–S lyase

We discovered an anaerobic C2 sulfonate C–S lyase candidate while carrying out a bioinformatics study on the glycyl radical enzyme (GRE) superfamily. GREs catalyze challenging chemical transformations using the high reactivity of the protein-based radical to initiate catalytic reactions^13^, and recent studies have yielded several new GREs with novel catalytic chemistry^13–15^. The essential O_2_-sensitive glycyl radical (G•) cofactor is generated by an activating enzyme through chemistry involving S-adenosylmethionine (SAM) and a [4Fe-4S]^1+^ cluster^16^.

To facilitate large-scale analysis of GRE sequences, a sequence similarity network (SSN)^17^ for 14228 unique sequences in the InterPro family IPR004184 (release 68.0) was constructed using the web-based Enzyme Function Initiative Enzyme Similarity Tool (EFI-EST)^18^, and displayed using Cytoscape v3.5^19^ at an E-value threshold of 10^–299^ (>∼60% sequence identity is required to draw an edge), which separates previously characterized GREs with mechanistically distinct catalytic activities into distinct clusters (Supplementary Fig. 2). While examining the remaining uncharacterized GRE clusters, we noticed a particular cluster containing a GRE of unknown function (GUF), dominated by sequences from phylogenetically diverse SSRB (Supplementary Fig. 2, Supplementary Data 1). Because most GREs are involved in fermentative rather than respiratory metabolism, the association of GUF with SSRB suggested a link to their unique mode of anaerobic respiration using sulfite as terminal electron acceptors (TEAs).

**Fig. 1:**
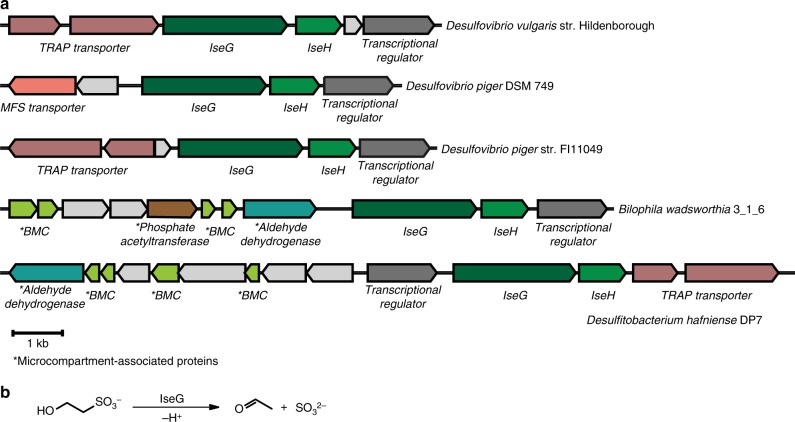
Genome neighborhood and proposed reaction. **a** The IseG genome neighborhoods in sulfate-reducing bacteria *Desulfovibrio vulgaris* str. Hildenborough, *Desulfovibrio piger* DSM749, *Desulfovibrio piger* str. F111049 (a gram-negative sulfate-reducing bacterium), *Desulfitobacterium hafniense* DP7 (a gram-positive sulfite-reducing bacterium) and sulfite-reducing bacterium *Bilophila wadsworthia* 3_1_6 (a gram-negative sulfite-reducing bacterium) are shown. In many cases, IseG is associated with a TRAP transporter and microcompartment proteins. **b** Proposed IseG-catalyzed reaction

In some SSRB, GUF is associated with microcompartment proteins^20^ (Fig. 1a), providing a clue regarding its potential substrate. Microcompartment-associated enzymes are often 1, 2-eliminases acting on substrates containing a C1-OH group and a C2 leaving group, generating acetaldehyde or propionaldehyde as products. These include the adenosylcobalamin-dependent enzymes diol dehydratase and ethanolamine-ammonia lyase (EAL)^21^, and the GREs choline-trimethylamine lyase (CutC)^22^, and propanediol dehydratase (PDH)^13^. GUF is also associated with a three-subunit TRAP transporter (Fig. 1a), a family of transporters that catalyze the import of organic acids and aliphatic sulfonates^23,24^. Taken together, we hypothesized that GUF acts on isethionate, catalyzing C–S cleavage to generate acetaldehyde and sulfite (Fig. 1b).

### GUF is an isethionate sulfo-lyase

**Fig. 2:**
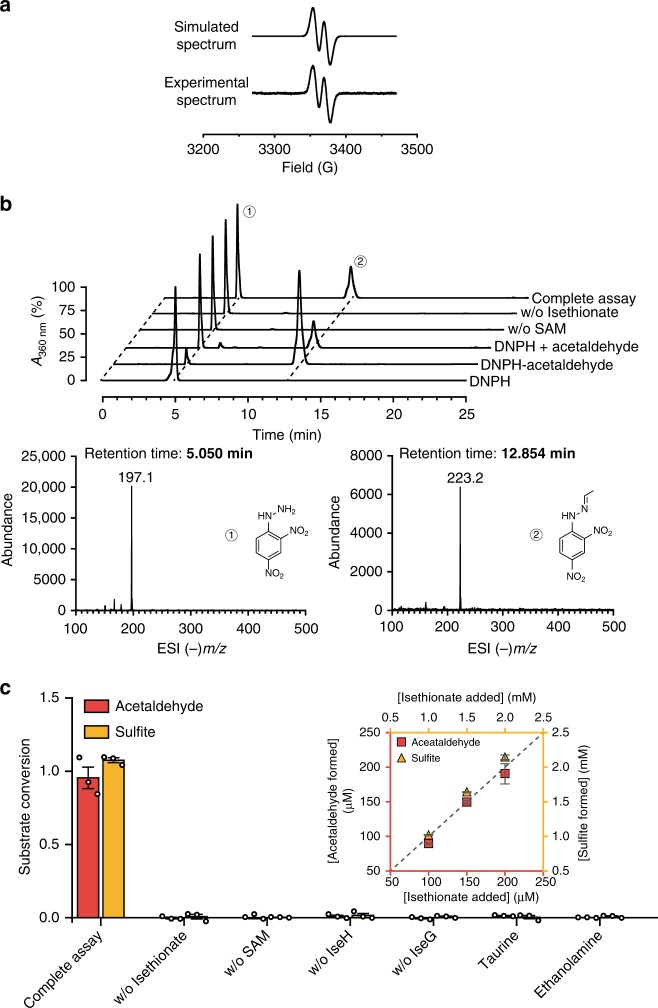
EPR spectra and enzymatic assays. **a** X-band EPR spectrum of IseG reconstituted with IseH and SAM in the presence of reductant (Ti (III) citrate). **b** Detection of acetaldehyde formation in the IseG-catalyzed isethionate cleavage using LC-MS/MS. HPLC elution profiles of the DNPH derivatives from the reaction products, reaction negative controls, and authentic standards (theoretical mass of the monoanion of DNPH = 197.1, and DNPH-acetaldehyde = 223.2), are presented. Negative ionization mass spectra of the peaks 1 and 2 from the HPLC trace show that they contain DNPH and DNPH-acetaldehyde, respectively. **c** Enzymatic assays showing the fraction of substrate converted to acetaldehyde and sulfite under different reaction conditions, demonstrating the reaction requirements and substrate specificity. The inset shows the stoichiometric conversion of isethionate into acetaldehyde and sulfite. The error bars represent the standard deviation of three individual experiments. Source data are provided as a Source Data file

To test this hypothesis, we recombinantly produced GUF (UniProt: Q727N1) and its adjacent activating enzyme (Q727N0) from the model SSRB *Desulfovibrio vulgaris* Hildenborough (Supplementary Fig. 3). Biochemical characterization confirmed that GUF was indeed an isethionate sulfo-lyase (Fig. 2, *vide infra*), and we renamed GUF and its activating enzyme IseG and IseH, respectively. Like most of the other GREs, the purified recombinant IseG exists as a dimer in solution, as analysed by size exclusion chromatography (SEC) (Supplementary Fig. 4).

Sequence alignments of IseH with other previously characterized GRE activating enzymes and ferredoxins from different origins show that, like many other activating enzymes, IseH contains the essential CX2CX3C motif that coordinates the radical SAM [4Fe-4S] cluster, as well as a widely conserved 8-cysteine motif in ferredoxin coordinating two [4Fe-4S] clusters (Supplementary Fig. 5a).^25^ Biochemical experiments and sequence analysis of hydroxyphenylacetate decarboxylase activating enzyme by Selvaraj et al.^25^ support this. Anaerobic reconstitution of the IseH [4Fe-4S] clusters resulted in 5.9 ± 0.1 Fe and 8.4 ± 0.1 S per monomer (out of the theoretical maximum of 12 Fe and 12 S) (Supplementary Fig. 5b), suggesting that our protocol gives rise to incomplete reconstitution and possibly a fraction of [3Fe–4S] clusters.^26^ IseH exhibited a typical UV-Vis spectrum for a [4Fe–4S] cluster-containing protein (Supplementary Fig. 6). Like other radical SAM enzymes, IseH cleaved SAM to form 5′-deoxyadenosine in the presence of a strong reductant Ti (III) citrate (Supplementary Fig. 7). Electron paramagnetic resonance (EPR) spectroscopy showed that IseH could install the G• on IseG, forming 0.4 (out of a theoretical maximum of 1)^16^ radicals per dimer (Fig. 2a).

Incubation of activated IseG with isethionate resulted in its conversion to acetaldehyde (Fig. 2b) and sulfite (Supplementary Fig. 8). Under conditions for full substrate conversion, the stoichiometry of isethionate added to acetaldehyde and sulfite produced was 1:1:1 (Fig. 2c). No activity was detected with taurine or ethanolamine as substrates, indicating high substrate specificity (Fig. 2c). The kinetic parameters of IseG support the physiological relevance of this reaction (*k*_cat_ = 91.6 ± 3.3 s^–1^, *K*_m_ = 44.8 ± 3.5 mM) (Supplementary Figs. 9, 10).

### Crystal structure of isethionate-bound IseG

**Fig. 3:**
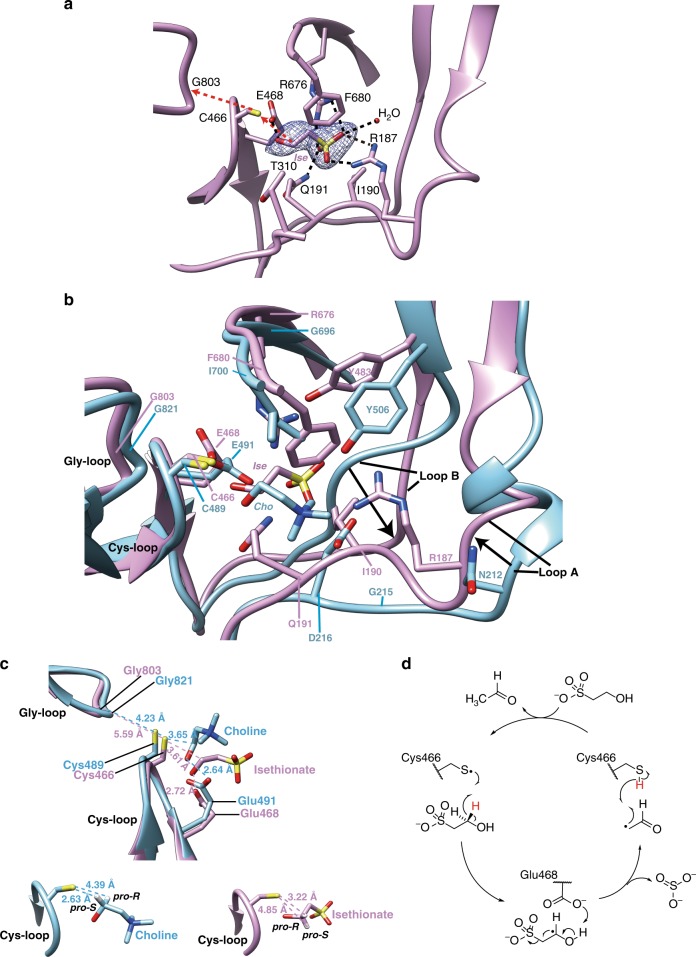
IseG active site structure. **a** IseG active site in complex with the substrate isethionate. The proposed pathway for hydrogen atom transfer is indicated by red arrows, and all hydrogen bonds are indicated by black dashed lines. 2Fo-Fc electron densities for isethionate are shown at 1.0σ. **b** Superposition of the IseG active site (plum) with CutC active site (cyan, PDB code 5FAU). Key residues involved in substrate binding and radical chemistry are displayed and labeled. The conformational changes in two substrate-coordinating loops are indicated by black arrows. **c** Structural model of the Gly-loop, Cys-loop, and the substrates. The distances between the key atoms are indicated. Comparison of the orientation of the modeled C1 hydrogens of isethionate (plum) and choline (cyan), suggesting that different enantiotopic hydrogens are abstracted by the thiyl radical. The distances between thiyl radical site and C1 hydrogens are labeled. **d** Proposed mechanism of isethionate cleavage by GUF (IseG). The thiyl radical, which is transiently generated by the G• cofactor in all GREs, abstracts a hydrogen (shown in red) from the substrate isethionate, and returns it to form the product acetaldehyde

To further investigate the mechanism of C–S cleavage by IseG, we determined the crystal structure of isethionate-bound IseG at 2.4 Å (Fig. 3, Supplementary Fig. 11). The P21 crystals contain four monomers per asymmetric unit, each exhibiting a canonical β/α barrel fold common to other GREs (Supplementary Fig. 11a). The superimposed structures of IseG and its closest structurally characterized relative CutC^22^ display a root mean square deviation (RMSD) of 1.25 Å between 503 Cα atoms. The positions of the isethionate C1 and OH groups relative to the glycyl and thiyl radical residues required for substrate activation, and a conserved glutamate required for deprotonation of the isethionate OH group (Fig. 3a–c) are consistent with our working hypothesis for the catalytic mechanism for C–S cleavage (Fig. 3d), which we propose based on analogies to the mechanism of other GRE C–O and C–N lyases^22,27^.

The orientation of the isethionate C2 and sulfonate-leaving group differs greatly from choline in CutC. A 98.5° rotation of the C1–C2 axis in isethionate vs. choline (Supplementary Fig. 11b) results in the *pro-R* hydrogen of isethionate being oriented for abstraction by the thiyl radical (Fig. 3c). The hydrogen of the opposite stereochemistry is thought to be abstracted in all other mechanistically related GREs: CutC, propanediol dehydratase, glycerol dehydratase, and ribonucleotide reductase^28^. Despite overall structural similarities between IseG and CutC, two substrate-interacting loops (loop A and B) in IseG are shifted by up to 6.8 Å relative to CutC (Fig. 3b, Supplementary Fig. 11c), resulting in dramatic changes to the binding pocket structure and substrate-coordinating residues.

The sulfonate group of isethionate is coordinated by Arg187, Gln191, and Arg676 of IseG and one water molecule through an extensive hydrogen bond network (Fig. 3a). The conformation of isethionate is further stabilized by van der waals contact with Phe680, Ile190, and Thr310. The three sulfonate-coordinating residues (Arg187, Gln191, and Arg676) are conserved in the sequences within the “IseG” cluster of the GRE SSN (Supplementary Data 1), but are absent in CutC and other GREs (Supplementary Fig. 12), supporting the proposal that the “IseG” cluster enzymes are involved in sulfonate degradation.

### Isethionate dissimilation in *D. piger*

Examination of the “IseG” cluster shows that IseG is present in diverse environmental and human-associated SSRB (Supplementary Data 1), and we next investigated the involvement of IseG in sulfonate degradation in these bacteria. Certain SSRB, including *Desulfovibrio spp*., *Desulfitobacterium spp*., and *Bilophila wadsworthia* have been reported to use isethionate as a TEA, generating acetate and H_2_S^29,30^. Among the sequenced strains, this ability correlates with the presence of IseG (Supplementary Table 1). A previously discovered pathway for isethionate degradation in non-SSRB environmental bacteria involves a membrane-bound flavin-dependent isethionate dehydrogenase (IseJ) and Xsc^31^ (Supplementary Fig. 1b). However, a BLAST search for IseJ revealed no homologs in SSRB, and not all isethionate-dissimilating SSRB contain Xsc (Supplementary Table 1), suggesting a distinct pathway for isethionate degradation in SSRB.

**Fig. 4:**
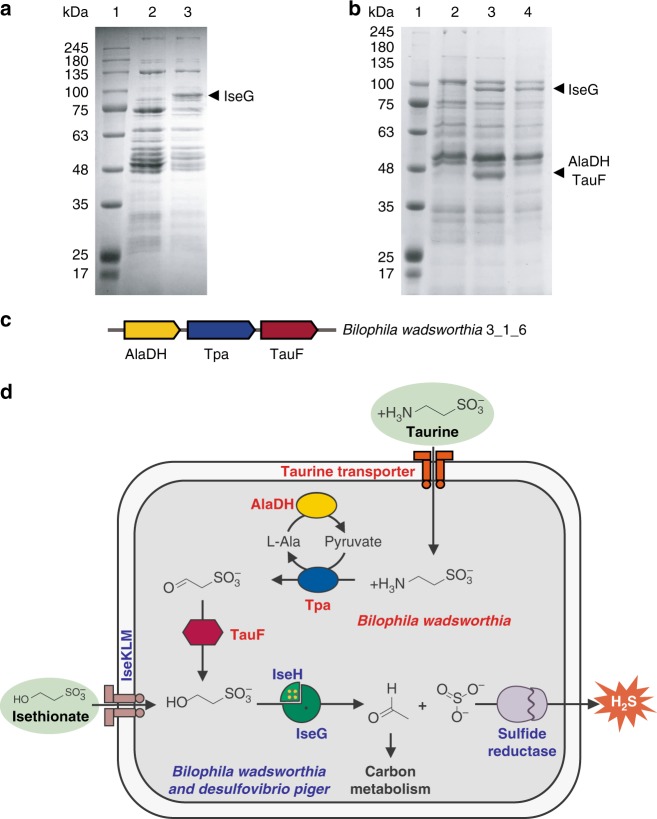
C2 sulfonate induced IseG-dependent pathway in SSRB. **a** SDS-PAGE analysis of *D. piger* grown on lactate plus sulfate (lane 2) or isethionate (lane 3). The arrow indicates a ~95-kD band identified as IseG. **b** *B. wadsworthia* grown on pyruvate plus thiosulfate (lane 2), taurine (lane 3) or isethionate (lane 4). The arrows indicate a ~95-kD band identified as IseG, and a ~40-kD band containing both AlaDH and TauF. **c** Genome neighborhood of *B. wadsworthia* Tpa and AlaDH, showing the proximity to a gene in the ADH superfamily, found to be a NADH-dependent sulfoacetaldehyde reductase TauF. **d** Proposed pathways for dissimilation of isethionate and taurine in *D. piger* and *B. wadsworthia*. Source data are provided as Supplementary Data 2–5

We further studied the most prevalent human gut sulfate-reducing bacterium *Desulfovibrio piger* DSM 749^32^, which contains IseG (Fig. 1a) but not Xsc (Supplementary Table 1). When lactate is supplied as the carbon and electron source, growth was supported with sulfate or isethionate, but not taurine, as the sole TEA. Growth was accompanied by the formation of a characteristic black FeS precipitate in the medium. This observation, together with the methylene blue assay for the headspace gas in the anaerobic culture vials, indicate H_2_S formation as previously reported for other *Desulfovibrio* species^2,30^ (Supplementary Fig. 13). SDS-PAGE analysis revealed a prominent protein band with molecular weight of ~95 kD (Fig. 4a), present in isethionate- but not sulfate-grown cells, as observed but not identified with several other SSRB by Leadbetter et. al. nearly 20 years ago^29^. Mass spectrometric analysis now identified this protein as IseG (B6WXM2, Supplementary Data 2).

We next investigated the mechanism for isethionate import. In *D. piger* DSM 749, IseG is associated with a major facilitator superfamily (MFS) transporter (Fig. 1a), a family of transporters that transport diverse substrates, which includes the putative isethionate MFS transporter IseU (Supplementary Fig. 1b)^31^. In other SSRB, including the *D. piger* strain FI11049, the IseG is associated with a TRAP transporter (Fig. 1a)^23^. The TRAP transporter family includes the putative isethionate TRAP transporter IseKLM, which is associated with other isethionate degradation pathways (Supplementary Fig. 1b)^31^. We chose to focus on the TRAP transporter as it is dependent on a soluble periplasmic substrate-binding subunit, which is amenable to biochemical characterization by isothermal titration calorimetry (ITC). The recombinant substrate-binding subunit (A0A1K1LFE7) of the IseG-associated TRAP transporter in *D. piger* strain FI11049 showed enthalpy-driven binding of isethionate with a physiologically relevant *K*_d_ of 0.5 μM (Supplementary Fig. 14a), but no binding of taurine (Supplementary Fig. 14b), confirming that it is an isethionate transporter. This pathway for uptake and dissimilation of isethionate (Fig. 4d) demonstrates a role for IseG in generating electron acceptors for anaerobic respiration.

### Isethionate and taurine dissimilation in *B. wadsworthia*

The disease-associated human gut sulfite-reducing bacterium *Bilophila wadsworthia* is one of a small number of SSRB reported to utilize taurine as the sole carbon and energy source for growth, generating acetate, ammonia and H_2_S^30^. It contains IseG (Fig. 1a), and we next investigated the involvement of IseG in taurine degradation in *Bilophila*. In the non-SSRB *Ruegeria pomeroyi* DSS-3, which has served as a paradigm for taurine dissimilation, taurine pyruvate aminotransferase (Tpa) converts taurine to sulfoacetaldehyde, followed by C–S cleavage by Xsc (Supplementary Fig. 1c)^7,33^. Pyruvate, required by Tpa, is regenerated by alanine dehydrogenase (AlaDH). In *B. wadsworthia* RZATAU, Tpa and AlaDH activities have been detected in lysates of taurine-grown cells, and their primary sequences have been identified^34,35^. However, Xsc activity could not be detected in the lysates^1^, and a BLAST search for Xsc revealed no homologs in both of the sequenced *Bilophila* species, suggesting a distinct pathway for taurine degradation in *Bilophila*.

In the genome of *B. wadsworthia* 3_1_6, Tpa occurs immediately downstream of AlaDH (Fig. 4c). Closer inspection revealed an uncharacterized metal-dependent alcohol dehydrogenase (Fe-ADH) (E5Y946) immediately downstream of Tpa. The recombinant protein, which we renamed TauF, catalyzed NADH-dependent reduction of sulfoacetaldehyde (Supplementary Fig. 15), generating isethionate that could subsequently be cleaved by IseG. This suggested a possible IseG-dependent taurine degradation pathway in *Bilophila* (Fig. 4d).

Growth of *B. wadsworthia* RZATAU was supported with pyruvate plus thiosulfate, or with either taurine or isethionate as the sole substrates, as previously reported^30^. Growth was accompanied by the formation of a characteristic black FeS precipitate in the medium, indicating H_2_S formation, as previously reported^30^. SDS-PAGE analysis revealed that prominent protein bands with molecular weight of ~95 kD (Fig. 4b), were present in isethionate- and taurine-grown cells but not thiosulfate-grown cells. Mass spectrometric analysis identified the proteins as IseG (E5Y378, Supplementary Data 3 and Supplementary Data 4). Taurine-grown cells also exhibited a prominent protein band with molecular weight of ~40 kD (Fig. 4b), identified as AlaDH and TauF (top two hits in Supplementary Data 5). This demonstrates that IseG-dependent pathways are responsible for the dissimilation of both isethionate and taurine in *Bilophila* (Fig. 4d), thus playing a critical role in the unique metabolic niche of this pathobiont, which relies on these sulfonates as TEAs in the gut environment^6^.

## Discussion

Discovery of the C–S lyase IseG adds to the chemical diversity of radical-dependent 1,2-lyases, which have hitherto included only C–O lyases (GRE and B12-dependent diol dehydratases) and C–N lyases (GRE CutC and B12-dependent EAL)^21,28^. The catalytic mechanisms of GRE and B12-dependent diol dehydratases have been studied in detail, both experimentally^36^ and computationally^27^, and are thought to proceed via different reaction mechanisms. In the proposed mechanisms, the reaction proceeds via a direct loss of the leaving group for GREs, and via a 1,2-shift of the leaving group for B12-dependent lyases^27,36^. Further biochemical and computational studies are clearly needed to investigate the C–S lyase reaction, to ascertain whether it may occur via one or both types of catalytic mechanism.

Our in vivo experiments with the two prominent human gut SSRB *D. piger* and *B. wadsworthia* demonstrate the involvement of the O_2_-sensitive IseG in C2 sulfonate dissimilation pathways in SSRB, and resolve the decades-old mystery of the mechanism of C–S cleavage in these strict anaerobes. Just as Xsc serves a hub in diverse pathways for C2 sulfonate degradation in many environmental bacteria, the O_2_-sensitive IseG may serve as a hub for alternative pathways for C2 sulfonate degradation in strict anaerobes, thereby serving as a genetic marker for the exploration of sulfonate-related biochemical pathways in the anaerobic microbiome.

IseG-dependent dissimilative pathways may play important roles in gut SSRB, highlighted by the presence of IseGH in all five SSRB genomes currently in the NIH Human Microbiome Project, including *Desulfitobacterium hafniense* DP7 (G9XK69), *D. piger* ATCC 29098 (B6WXM2), *Desulfovibrio* sp. 6_1_46AFAA (G1UPG5), *B. wadsworthia* 3_1_6 (E5Y378), and *Bilophila* sp. 4_1_30 (G1V3L3). In addition, the IseG-dependent pathway is the only known pathways for sulfonate degradation in *D. piger* and *B. wadsworthia*. The abundance of SSRB correlates with many human diseases^37,38^, and have been shown to alter the levels of H_2_S^39^, a signaling molecule in plasma and many tissues^40^, with significant effects on neuronal activity and behavior^41^. Therefore, pathways involving IseGH, will impact future microbiome studies and present therapeutic targets for the treatment of human diseases.

## Methods

### Materials and general methods

Lysogeny Broth (LB) medium was prepared with yeast extract and tryptone purchased from Oxoid, England. Anaerobe Basal Broth (ABB) rich medium was purchased from Rishui Biotech., Qingdao, China. Methanol and acetonitrile used for liquid chromatography-mass spectrometry (LC-MS) were high-purity solvents from Concord Technology. Formic acid was purchased from Merck. Water used in this work was ultrapure deionized water from Millipore Direct-Q. Oligonucleotide primers were synthesized by General Biosystems, Inc (Supplementary Table 2). Talon Cobalt resins were purchased from Clonetech. All other reagents unless specified were purchased from Sigma/Aldrich.

All protein purification chromatographic experiments were performed on an “ÄKTA pure” or “ÄKTA prime plus” FPLC machine equipped with appropriate columns (GE Healthcare). Protein concentrations were calculated from the absorption at 280 nm measured using an Eppendorf BioPhotometer D30 or determined by Bradford assays referring to the standard curves established with a series of concentrations of bovine serum albumin (BSA). Anaerobic experiments were conducted in a Lab2000 glovebox (Etelux) under an atmosphere consisting of N_2_ with less than 5 ppm O_2_.

### Plasmid construction

DNA fragments containing codon-optimized ORFs of IseG, IseH, *Dp*IseK and *Bw*TauF were synthesized and inserted into plasmids by General Biosystems, Inc (Supplementary Data 6). Plasmids used in this study were pET-28a(+) and modified pET28 vectors including HMT vector (containing, in tandem, a His_6_-tag, maltose binding protein (MBP) and a Tobacco Etch Virus (TEV) protease cleavage site, followed by the construct of interest), and HT vector (containing a His_6_-tag and a TEV protease cleavage site, followed by the construct of interest)^42^. For the production of IseG for biochemical studies, the *IseG* fragment was inserted into pET-28a(+) at the *Nde*I/*Xho*I sites to form pET-28a(+)-His_6_-IseG. For the production of soluble IseH, the *IseH* fragment was inserted into the HMT vector at the *Ssp*I site. For the production of high purity IseG with an N-terminal 23-a.a truncation for crystallography, the *IseG* fragment was amplified by PCR using primers 1F and 1R (Supplementary Table 2) and inserted into the HMT vector at the *Ssp*I site by Gibson assembly to form HMT-IseG(−23aa). A surface-entropy reduction mutation, changing a.a. 133–136 from EDAR to AAAA, was introduced by QuickChange site-directed mutagenesis using primers 2F and 2R (Supplementary Table 2) to form pET28-HMT-IseG(−23aa 133EDAR136-AAAA). The *DpIseK* fragment was inserted into the HMT vector at the *Ssp*I site for the expression of recombinant MBP-IseK fusion protein and subsequent ITC experiment. The *BwTauF* fragment was inserted into the HT vector at the *Ssp*I site.

### Recombinant protein production and purification

IseG and MBP-IseH were heterologously expressed in *Escherichia coli* BL21 (DE3) cells harboring the plasmids pET-28a(+)-His_6_-IseG and HMT-IseH, respectively. For IseH, the cells were co-transformed with the plasmid pTf16 (TaKaRa) for co-expression of the tig chaperone. For IseG expression, LB containing 50 μg/mL kanamycin was used, while for MBP-IseH, LB containing 50 μg/mL kanamycin and 25 μg/mL chloramphenicol was used. Single colonies were inoculated into 5 mL starter cultures, grown at 37 °C overnight, and transferred into 1 L of medium. For MPB-IseH, 0.5 mg/mL l-arabinose was added to induce expression of the chaperone. The cultures were grown at 37 °C with shaking at 220 rpm. When OD_600_ reached ∼0.8, the temperature was decreased to 16 °C and isopropyl β-d-1-thiogalactopyranoside (IPTG) was added to a final concentration of 0.3 mM. Cells were harvested by centrifugation (4000 × *g* for 10 min at 4 °C) after 16 h^14^. Cells (~1 g wet weight) were suspended in 5 mL of lysis buffer [50 mM Tris/HCl, pH 8.0, 1 mM phenylmethanesulfonyl fluoride (PMSF), 0.2 mg/mL lysozyme, 0.03% Triton X-100, and 1 μL of DNase I (Roche)]. The cell suspension was frozen in a −80 °C freezer, and then thawed and incubated at room temperature (RT) for 50 min to allow lysis. 15 mL of buffer A [20 mM Tris/HCl, pH 7.5, and 5 mM β-mercaptoethanol (BME)] containing 1.3% streptomycin sulfate was added, and the precipitated DNA was removed by centrifugation (20,000×*g* for 5 min at 4 °C). Solid (NH_4_)_2_SO_4_ was then added to 70% saturation. The solution was shaken for an additional 10 min, and the precipitated protein was isolated by centrifugation (20,000×*g* for 10 min at 4 °C). The pellet was dissolved in 10 mL of buffer B (buffer A containing 0.2 M KCl), filtered and applied to a 2 mL TALON (Clontech) gravity column, pre-equilibrated with buffer B. The column was washed with 10 column volumes (CV) of buffer B, and protein was eluted with 5 CV of buffer B containing 150 mM imidazole. The eluted protein was precipitated with solid (NH_4_)_2_SO_4_ to 70% saturation and isolated by centrifugation (20,000 × *g* for 10 min at 4 °C). The pellet was dissolved in 0.5 mL of buffer B and desalted using a G25 column (GE, thermostat jacket tube XK16/20, packed 15 cm × 2 cm^2^, 30 mL), pre-equilibrated with buffer C [20 mM Tris/HCl, pH 7.5, 100 mM KCl, 10% glycerol, and 1 mM tris(2-carboxyethyl)phosphine (TCEP) for IseG or 1 mM dithiothreitol (DTT) for MBP-IseH]^14^. The eluted protein was concentrated to ~400 μL by ultrafiltration (Sartorius VIVASPIN TURBO 15 (30,000MWCO), frozen in aliquots in liquid N_2_, and stored at −80 °C. The purified IseG (*ε*_280_ = 129,150 M^−1^ cm^−1^) and MBP-IseH (*ε*_280_ = 93,740 M^−1^ cm^−1^) were examined by SDS-PAGE on a 12% gel.

Details for recombinant production and purification of the other proteins, including IseG (−23 a.a.), *Sc*ADH, *Bw*IseK, and *Bw*TauF, are provided in the Supplementary Methods. MBP-IseH was used for biochemical experiments without cleavage of the MBP tag. The MBP tag of MBP-*Dp*IseK was cleaved and removed prior to ITC experiments.

### Analysis of the oligomeric state of IseG

A 2.5 mg/mL solution of IseG (−23 a.a.) was analysed by gel filtration. A 2 mL protein solution was injected into a Superdex 200 gel filtration column (300 mL) and eluted over 170 min with buffer D (10 mM HEPES/KOH, pH 7.4, 250 mM KCl, 10 mM BME) at 3 mL/min. The same conditions were used to analyze a mixture of molecular weight markers. The molecular weights of the proteins were calculated from their elution volumes, using a second-degree polynomial for the relationship between log(molecular weight) and retention time. The observed molecular weight for IseG (−23 a.a.) was 196 kDa, whereas the calculated molecular weight for IseG monomer is 91.3 kDa. This result indicates that IseG exists as a dimer in solution, which is consistent with the oligomeric state of pyruvate formate-lyase and other GREs studied to date^43^.

### [Fe-S] cluster reconstitution for MBP-IseH

A solution of MBP-IseH (50 μM) was degassed on a Schlenk line and brought into the glovebox. The reconstitution buffer contained 10 mM DTT and 100 mM Tris-HCl, pH 7.5. A solution of ferrous ammonium sulfate (12 eq.) was added followed by a solution of sodium sulfide (12 eq.). The mixture was incubated overnight at 4 °C in a cooling-heating block (Dry Bath H2O3–100C, Coyote Bioscience, Beijing, China). A solution of EDTA (12 eq.) was then added, and excess of iron and sulfide removed by repeated concentration with a centrifugal filter unit (1.5 mL YM-30 Amicon, Millipore), and dilution with buffer containing 20 mM Tris-HCl, pH 7.5 and 0.1 M KCl.

Ferrozine (3-(2-pyridyl)−5,6-diphenyl-1,2,4-triazine-p,p′-disulfonic acid monosodium salt) assays were carried out to quantitate iron contents in as-isolated and reconstituted MPB-IseH proteins. The standard curve was established with “Iron standard for AAS” (Fluka catalog # 16596) in a range from 0–600 μM. Briefly, 50 μL protein sample was mixed with 100 μL 2 M HCl. The protein was denatured in a boiling water bath for 10 min. The solution was then centrifuged for 5 min to remove precipitated protein. After cooling to RT, 150 μL saturated ammonium acetate, 150 μL freshly prepared 10 mM sodium ascorbate, and 200 μL 10 mM ferrozine were added and mixed. 200 μL of the mixture was transferred to a 96-well plate for the measurement of *A*_562_ with a Tecan M200 plate reader and the reading was referred to the standard curve for iron quantitation. The sulfide contents of as-isolated and reconstituted MBP-IseH were determined by measuring the absorbance of methylene blue formed upon reaction with *N*,*N*-dimethyl-p-phenylenediamine dihydrochloride (DPD)^44,45^. The purity of MBP-IseH was estimated to be 50% based on densitometry analysis of the Coomassie-stained SDS-PAGE gel using the software ImageJ and used to estimate the Fe and S contents per IseH monomer.

### UV-Vis absorption spectra of the reconstituted MBP-IseH

A solution of reconstituted MBP-IseH was diluted to 10 μM with a buffer containing 20 mM Tris/HCl, pH 7.5, 100 mM KCl. Samples were transferred into a septum-sealed anaerobic cuvette (Starna Cells, Quartz Septum Cell), taken out of the glovebox and the absorption spectrum acquired in the 200–800 nm range using a Hitachi U3900 spectrometer. To obtain the absorption spectrum of reduced IseH, 10 equivalents of Ti(III) citrate or 100 equivalents of sodium dithionite (NaDT) was injected using a Hamilton air-tight syringe and incubated for 5 or 15 min respectively prior to the absorbance measurement. The UV-Vis absorption spectra exhibited features characteristic of [4Fe-4S]^2+^ clusters, which disappeared upon reduction. Correcting for the 50% purity of MBP-IseH, the extinction coefficient of the reconstituted MBP-IseH [4Fe-4S] clusters was estimated to be 38 mM^−1^ cm^−1^. Given the approximate *ε*_410nm_ of 15 mM^−1^ cm^−1^ per cluster^46^, we estimated that ~2.5 [4Fe-4S] clusters per monomer were reconstituted, consistent with the measured Fe and S contents.

### LC-MS assays for SAM cleavage by IseH

Detection of IseH catalyzed SAM cleavage and product formation using LC-MS assays were performed as described elsewhere^47^. A reaction mixture (500 μL total volume) containing 20 mM Tris/HCl, pH 7.5, 100 mM KCl, 200 μM Ti(III) citrate, and 20 μM reconstituted MBP-IseH was incubated for 15 min at RT in the glovebox to allow the reduction of IseH. SAM (Sigma, 0.5 mM final concentration) was added to initiate the cleavage reaction. A control assay omitting Ti (III) was also performed. The reaction was incubated at RT in the glovebox overnight and quenched with formic acid (5% v/v final concentration). The reaction mixture was then incubated in a boiling water-bath for 45 s to completely denature the protein. The precipitated protein was removed by centrifugation at 14,000 × *g* for 10 min. and the supernatant was filtered through a 0.22-μm PES membrane. A 20 μL portion of the supernatant was analyzed by an Agilent 6420 Triple Quadrupole LC/MS instrument (Agilent Technologies) on a C18 column (Advantage ECHELON C18 4 μm 150 × 2.1 mm P/N: ADV8010, manufactured by ANALYTICAL). The solvent system consisted of water (A) and acetonitrile (B), and the sample was eluted with a linear gradient of 0–16% B over 30 min, with a flow rate of 0.5 mL/min. The products were detected by UV absorption at 257 nm, and 5′-dA was compared to commercial standard and verified by mass spectrometry.

### EPR detection of IseG glycyl radical formation

Continuous wave X-band electron paramagnetic resonance (EPR) spectroscopy was used to characterize the IseG glycyl radical. A 240 μL reaction mixture containing 20 mM HEPES, pH 7.5, 0.1 M KCl, 20 μM IseG, 80 μM reconstituted MBP-IseH, 1 mM SAM, 100 μM Ti(III) citrate and 5% glycerol was incubated at RT for 10 min in the glovebox. A control sample omitting Ti(III) citrate was also prepared. All samples were loaded into EPR tubes with 4 mm o.d. and 8″ length (Wilmad Lab-Glass, 734-LPV-7), sealed with a rubber stopper, removed from the glovebox and frozen in liquid nitrogen prior to EPR analysis. The perpendicular mode X-band EPR spectra were recorded using a Bruker E500 EPR spectrometer. Data acquisition was performed with Xepr software (Bruker). The EPR spectra represent an average of 30 scans and were recorded under the following conditions: temperature, 90 K; center field, 3370 Gauss; range, 200 Gauss; microwave power, 10 μW; microwave frequency, 9.44 MHz; modulation amplitude, 0.5 mT; modulation frequency, 100 kHz; time constant, 20.48 ms; conversion time, 30 ms; scan time, 92.16 s; and receiver gain, 43 dB. The experimental spectra for the glycyl radical were modeled with Bruker Xepr spin fit to obtain g values, hyperfine coupling constants, and line widths. Double integration of the simulated spectra was used to measure spin concentration.

### Fuchsin assay for sulfite detection

Sulfite was detected using a colorimetric assay involving the formation of a colored complex between sulfite and fuchsin dye in acidic solution^48^. IseG activation was carried out as described for the EPR experiments except that glycerol was omitted. A 100 µL reaction mixture containing 10 μM activated IseG, 10 μM *Sc*ADH1, 1 mM NADH, 600 μM isethionate (Adamas) was incubated at 30 °C for 1 h in the glovebox. A negative control omitting isethionate was also performed. While the reaction incubated, stock solution A (0.8 M H_2_SO_4_, 0.08% Fuchsin and 1.6% formaldehyde, mixed 7: 2: 1) was freshly prepared. A 50 µL portion per reaction sample was mixed with 950 µL of solution A, incubated for 10 min, and the UV-Vis absorption spectra were collected.

### LC-MS assay for acetaldehyde detection

A 100 μL reaction mixture containing 10 μM IseG, 40 μM IseH, 0.05 mM Ti(III) citrate, 0.5 mM SAM, and 10 mM isethionate was incubated at 30 °C for 20 min in the glovebox. Two negative controls omitting either SAM or isethionate were also performed. The acetaldehyde product was detected by derivatization with 2,4-dinitrophenylhydrazine (DNPH) (J&K)^49^. After the enzyme reaction, 100 μL of reaction solution was mixed with 1.1 mL of 0.73 M sodium acetate buffer pH 5.0, followed by 800 μL of freshly prepared DNPH solution (40 mg dissolved in 100 mL methanol), and the mixture was incubated at 50 °C for 1 h and then filtered prior to LC-MS analysis. A commercial acetaldehyde-DNPH standard (Sigma) (40 mg in 250 mL 40% methanol and 60% H_2_O) was also prepared.

LC-MS analysis was performed on an Agilent 6420 Triple Quadrupole LC/MS instrument (Agilent Technologies). The drying gas temperature was maintained at 350 °C with a flow rate of 12 L min^–1^ and a nebulizer pressure of 25 psi. LC-MS analysis was carried out with 20 μL sample volume on an Agilent ZORBAX SB-C18 column (4.6 × 250 mm, product number 880975–902). The column was equilibrated with 50% H_2_O/50% CH_3_CN and developed at a flow rate of 1.0 mL/min. UV detection was set at 360 nm. The LC elution profile for the complete assay contained two major peaks with retention times of 5.1 and 12.9 min corresponding to DNPH and acetaldehyde-DNPH, respectively, as identified by ESI-MS (m/z) and by comparison to the respective standards. The acetaldehyde-DNPH peak was absent in both negative controls. Taken together, these observations demonstrate that IseG catalyzes the cleavage of isethionate to form acetaldehyde.

### Spectrophotometric coupled assay for acetaldehyde formation

Assays involving complete substrate turnover were conducted to determine the stoichiometry of substrate consumed to products formed. An NADH-coupled spectrophotometric assay was used to quantitate acetaldehyde formation. A 100 µL reaction mixture containing 10 µM activated IseG, 10 µM *Sc*ADH1, 0.3 mM NADH and 100, 150, or 200 µM isethionate was incubated at RT for 40 min in the glovebox. Control assays omitting either isethionate, SAM, IseH or IseG were also performed. Assays in which isethionate was substituted with 200 µM taurine (Solarbio) or ethanolamine (Adamas) were also performed to examine the substrate specificity of IseG. The decrease of *A*_340 nm_ for samples contained in a 1-cm cuvette was monitored using a Thermo Scientific Nanodrop OneC machine (cuvette mode) in the glovebox and used to calculate the substrate turnover.

### DTNB assay to quantitate sulfite formation

DTNB (5,5-dithio-bis-(2-nitrobenzoic acid), Ellman’s Reagent) was used to quantitate sulfite formation in the complete turn over assays^50^. *Sc*ADH1 and NADH were omitted in these assays. Since the proteins, residual DTT and metals can interfere with the DTNB assays, higher isethionate concentrations were used. A 40 µL reaction mixture containing 10 µM activated IseG and 1, 1.5, and 2 mM isethionate was incubated at RT for 1 h in the glovebox. 40 µL of 3 mM EDTA and 120 µL of 0.1 M phosphate buffer pH 8.0 were then added and mixed. 70 µL of the mixture was then added to 30 µL freshly prepared DTNB solution (5 mM in 0.1 M phosphate buffer, pH 8.0) and subjected to *A*_412 nm_ measurement. Sulfite formation was calculated by referring to a standard curve established with known concentrations of sulfite spiked into the same reaction mixture omitting isethionate.

### Kinetic assays

The spectrophotometric coupled kinetic assays (assay volume 100 µL) for IseG activity were conducted at RT in a 1 cm Eppendorf cuvette using the cuvette mode of the Thermo Scientific Nanodrop OneC in the glovebox. The absorbance of each assay mixture was monitored at 340 nm, at 2 s intervals. For enzyme dose-dependent assays, the enzyme concentration was varied while a fixed, saturating substrate concentration of 400 mM isethionate was used. To obtain the Michaelis-Menten kinetic parameters, assays were performed with varied substrate concentrations and a fixed enzyme concentration of 250 nM IseG. GraphPad Prism6 was used for data analysis.

### X-ray crystal structure of IseG

Initial screening of IseG crystals was performed using an automated liquid handling robotic system (Gryphon, Art Robbins) in 96-well format by the sitting-drop vapor diffusion method. The screens were set up at 295 K using various sparse matrix crystal screening kits from Hampton Research and Molecular Dimensions. Several crystallization conditions gave thin plate-shape crystals. After further optimization using the hanging-drop vapor-diffusion method in 24-well plates with protein concentration at 10 mg/mL, we obtained crystals large enough for single crystal X-ray diffraction studies. The best condition yielding large plate crystals was 0.2 M sodium malonate dibasic monohydrate, 0.1 M Bis-Tris propane, pH 8.5, 20% (w/v) PEG3350 plus 500 mM isethionate.

Crystals were flash-cooled in liquid nitrogen using reservoir solution containing 30% glycerol as cryoprotectant. Diffraction data was collected on a local Rigaku X-ray diffractor (XtaLAB P200 MM007HF) to a resolution of 2.40 Å. The data set was indexed, integrated and scaled using HKL3000 suite^51^. Molecular replacement was performed by PHENIX^52^ using a homology model of IseG created by Phyre2 server^53^. The structure was manually built according to the modified experimental electron density using Coot^54^ and further refined by PHENIX^52^ in iterative cycles. Supplementary Table 3 contains the statistics for data collection and final refinement. All structural figures were generated with UCSF Chimera^55^.

### Growth of SSRB with different terminal electron acceptors

*Desulfovibrio piger* (DSM 749) was purchased from DSMZ, and cultured to investigate whether it can utilize taurine or isethionate as TEA. Cells were first inoculated into DSM641 defined medium and cultivated anaerobically at 37 °C for 2 days. Then 100 μL portions of the starter culture were transferred into three anaerobic bottles containing 5 mL modified DSM 641 defined medium, omitting Na_2_SO_4_ and with MgSO_4_ replaced by MgCl_2_. Different TEAs (20 mM) were added: (1) Na_2_SO_4_, (2) taurine, and (3) sodium isethionate. After 27 h incubation at 37 °C, the cultures containing Na_2_SO_4_ and isethionate, but not the one containing taurine, became turbid and black, indicating bacterial growth and formation of iron sulfide precipitates as a result of sulfate/sulfite reduction.

*Bilophila wadsworthia* (DSM 11045) was purchased from DSMZ. Cells were first inoculated into ABB medium supplemented with 5 mM taurine and cultivated anaerobically at 30 °C for 3–7 days. Then 100 μL portions of the starter culture were transferred into three anaerobic bottles containing 5 mL modified DSM 503 medium, omitting taurine and supplemented with 60 mM Na-formate and 200 µg/L 1,4-naphthochinone. Different TEAs (20 mM) were added: (1) Na_2_S_2_O_3_ (with 20 mM sodium pyruvate added as a carbon and electron source), (2) taurine, and (3) isethionate. After 3–7 days incubation at 30 °C, all three cultures became turbid and contain black precipitate, indicating bacterial growth and FeS formation.

### Detection of H_2_S formation in SSRB cell culture

*D. piger* was inoculated into 20 mL anaerobic starter culture containing DSMZ_Medium 641. 20 μL starter culture was transferred into two anaerobic vials containing 5 mL modified DSM 641 medium omitting SO_4_^2−^ and S^2−^ salt. 20 mM isethionate was added to one of the two vials. After 3 days’ incubation at 37 °C, the culture supplemented with isethionate exhibited turbidity with bacterial growth and black precipitate, while the culture without isethionate remained clear. From both vials, 1 mL headgas was taken out using a syringe, and introduced slowly into the liquid in an Eppendorf tube, containing 800 μL of 0.75% zinc acetate and 50 μL of 7% NaOH. The mixture was incubated at room temperature for 15 min. 150 μL of 0.1% DPD (1 mg DPD dissolved in 1 mL 5 N HCl) and 150 μL of 10 mM FeCl_3_ (dissolved in 1 N HCl) were added to the reaction mixture. The reaction mixture was incubated at 30 °C for 20 min and centrifuged at 14,000 × *g* for 5 min. The supernatant was subjected to absorbance measurement at 670 nm using a Tecan M200 plate reader.

### Protein identification by SDS-PAGE and mass spectrometry

Cells were harvested by centrifugation, lysed by boiling in Laemmli loading buffer, and analysed on a 10% SDS-PAGE gel. Prominent protein bands induced by growth on sulfonate substrates were manually excised and sent to Beijing Proteomic Research Centre for analysis. After in-gel digestion and extraction, the peptide mixtures were loaded onto LCESI-Q-TOF MS. The peptide hits searched against the *Desulfovibrio piger* protein database GCF_900116045.1 and GCF_000156375.1 (ATCC 29098), or the *Bilophila wadsworthia* protein database GCF_000185705.2 (Bilo_wads_3_1_6_V2) and GCF_000701705.1 (ATCC 49260), by MASCOT, Protein identifications were performed based on probability-based Mowse scoring algorithm with a confidence level of 95%.

For *D. piger*, isethionate-grown cells, but not sulfate-grown cells, exhibited a prominent protein band migrating at 95 kDa. This band was identified as glycyl radical protein from *Desulfovibrio piger* WP_006008826.1 and WP_072335172.1, corresponding to IseG (Supplementary Data 2). For *B. wadsworthia*, taurine- and isethionate-grown cells, but not the Na_2_S_2_O_3_ grown cells, exhibited a protein band at 95 kDa. Cells grown on taurine also exhibited a prominent protein band with molecular weight of ~40 kD. The 95 kDa band was identified as glycyl radical protein from *Bilophila wadsworthia* WP_005024906.1, corresponding to IseG (Supplementary Data 3 and 4). The 40 kDa band was identified as AlaDH and TauF (top two hits in Supplementary Data 5).

### ITC assays for *Dp*IseK

ITC measurements were performed on a PEAQ-ITC instrument (Malvern). The buffer for all of the experiments was 20 mM HEPES, pH 8.0 with 10 mM NaCl. Titrations consisted of 13 injections of 2 μL of 130 μM isethionate or taurine into the cell containing 50 μM *Dp*IseK or control buffer. The reference cell contained deionized water. Experiments were performed at 25 °C and a stirring speed of 750 rpm. Background titrations measuring the nonspecific heat released by the dilution of the ligands in the absence of protein were obtained and subtracted from the raw titration. The datasets were analysed using a single-site binding model using the MicroCal PEAQ-ITC software. Fitting was performed to derive stoichiometry, binding affinity, and changes in enthalpy (ΔH) and entropy (ΔS). A *K*_d_ of 0.5 μM was measured for isethionate. The *N* value is estimated to be 0.25, indicating sub-stoichiometric binding possibly due to a fraction of pre-bound or inactive protein. No interaction was observed for taurine.

### Enzyme activity assay for *Bw*TauF

Sulfoacetaldehyde is unstable and required to be introduced as a bisulfite adduct^56^. A 200 μL mixture containing 0.1 M Tris, pH 7.5, 0.1 M KCl, 5 mM sulfoacetaldehyde, 0.5 mM NADH was pre-mixed in a 96-well plate, followed by addition of 0.5 μg *Bw*TauF to initiate the reaction. Absorbance at 340 nm was monitored for 1.5 min at 15 s intervals. Negative controls omitting either sulfoacetaldehyde or *Bw*TauF were included. The time-dependent decrease of *A*_340_ in the complete assay but not in any of the negative controls demonstrates that *Bw*TauF is a sulfoacetaldehyde reductase.

### Reporting summary

Further information on experimental design is available in the Nature Research Reporting Summary linked to this article.

## Supplementary information

Supplementary Information

Peer Review File

Description of Additional Supplementary Files

Supplementary Data 1

Supplementary Data 2

Supplementary Data 3

Supplementary Data 4

Supplementary Data 5

Supplementary Data 6

Reporting Summary

Source Data

## Data Availability

Coordinates and structure factors of IseG in complex with isethionate have been deposited in the Protein Data Bank with accession code 5YMR. Source data underlying Fig. 2a–c, Supplementary Fig. 3–10, 13–15 are provided as a Source Data file. Source data underlying Supplementary Fig. 2 and Figs. 4a, 4b are provided as Supplementary Data 1–5. Other data are available from the corresponding authors upon reasonable request.
