# Supplementary material for: Radical-mediated C-S bond cleavage in C2 sulfonate degradation by anaerobic bacteria
Source: Nat Commun. 2019 Apr 8;10:1609. doi: 10.1038/s41467-019-09618-8 (PMC6453916; doi:10.1038/s41467-019-09618-8)
Supplement: Supplementary file 1 — Supplementary Information [file 41467_2019_9618_MOESM1_ESM.pdf]

Supplementary Information

Radical-mediated C-S bond cleavage in C2 sulfonate degradation by anaerobic bacteria

Xing et al.

Supplementary Methods

Expression of a MBP-IseG mutant for crystallography was carried out in *E. coli* BL21 (DE3) cells harbouring the plasmid pET28-HMT-IseG(-23aa 133EDAR136-AAAA). The transformant was grown in LB medium containing kanamycin (50 μg/mL) at 37°C in flasks in a shaker incubator at 240 rpm, and induced for the expression of IseG with 0.3 mM IPTG for 16 h at 20°C. Typically cells from 3 L culture were harvested by centrifugation (8000×*g* for 10 min) and lysed with French press (Panda plus, Niro Soavi Co., Italy) at 14,000 psi in 100 mL buffer A with 25 μg/mL DNaseI and 1 mM PMSF. The lysate was then centrifuged (40,000×*g* 30 min at 4°C) to remove unbroken cells and cell debris. The protein solution was then applied to a 10 mL Ni-NTA high-trap column. The column was washed with buffer A containing 20, 50 and 100 mM imidazole, 5 CV at each concentration. The protein was eluted with buffer A containing 150 mM imidazole. The eluate was then diluted two-fold with deionized water and loaded on a column packed with 40 mL amylose resins (New England BioLabs). The column was then washed with 5 CV buffer A and eluted with 5 CV buffer A containing 10 mM maltose. The eluate pre-mixed with 2.8 mg recombinant His_6-tagged TEV protease was dialyzed overnight against 5 L buffer A. The dialyzed sample was loaded on a 10 mL Ni-NTA column to retain TEV protease and MBP proteins. The flow-through was collected and dialyzed against 5 L buffer E (10 mM Tris-HCl, pH 8.8, 10 mM BME) for 3 h before it was applied to a 5 mL Q sepharose high performance column. The column was eluted with 10 CV linear salt gradient from 150 to 500 mM KCl in buffer E. A prominent peak containing IseG was collected and concentrated to a final volume of 2 mL (2.5 mg/mL) using a centrifuge concentrator (30K MWCO; Sartorius). This protein solution was then injected into a Superdex200 gel filtration column (300 mL) and eluted with buffer A. The eluate from gel filtration column was re-concentrated and buffer-exchanged with the storage buffer (10 mM HEPES, pH 7.4, 50 mM KCl, 1 mM TCEP). The final concentration is 10 mg/mL. The purified protein was examined by SDS-PAGE on a 12% gel.

The gene *Sc*ADH1 was amplified by PCR from *Saccharomyces cerevisiae* genomic DNA, using the primer pair 3F/3R (Supplementary Table 2). The PCR products were inserted into a customized expression vector (encoding an N-terminal MGSSHHHHHHSQSGSG tag) by Gibson assembly to form pACYC-*Sc*ADH1.

Expression of *S. cerevisiae* ADH1 was carried out in BL21 (DE3) *E. coli* cells harbouring the plasmid pACYC-*Sc*ADH1, using a protocol similar to that for IseG, except that the culture medium was LB containing 50 μg/mL chloramphenicol and 50 μM $ZnCl_2$ was added to the media during induction. *Sc*ADH1 was purified using a protocol similarly to that for IseG, except that the reductant (BME / DTT) was omitted from the purification and storage buffers. Purified *Sc*ADH1 was then examined by SDS-PAGE on a 12% gel. The activity of purified *Sc*ADH1 was measured in 50 mM sodium pyrophosphate buffer, pH 9.2, containing 16.7 ng *Sc*ADH1, 7.5 mM NAD^+ and 3.3% (v/v) ethanol as a substrate using a plater reader (Tecan M200) to monitor the increase of absorbance at 340 nm. The specific activity was 300 Unit/mg under this assay condition.

Clustal Omega[1] was used for multiple sequence alignment of IseH and other previously characterized GRE activating enzymes and ferredoxin of different origins to demonstrate conserved motifs containing cysteines as ligands for the active site [4Fe-4S] cluster and two auxiliary [4Fe-4S] clusters in a ferrodoxin-like domain.

Using the information obtained from our crystal structure and the structures of other GREs in the protein databank, structure-based sequence alignments were performed. Key residues involved in radical chemistry and substrate interaction were highlighted.

The recombinant HMT-*Dp*IseK plasmid was transformed into BL21 (DE3) cells. The transformant was grown in LB medium containing kanamycin (50 µg/mL) at 37°C in flasks in a shaker incubator at 220 rpm, and induced for the expression of MBP-IseK fusion protein with 0.4 mM IPTG for overnight at 18°C. The cells were harvested by centrifugation at 8000 g for 10 min.

MBP-*Dp*IseK was purified using a 5 mL home-packed TALON Cobalt gravity column. To cleave the MBP tag, the eluate from the TALON column, pre-mixed with recombinant His_6-tagged TEV protease (estimated TEV protease to MBP-*Dp*IseK at 1:5 molar ratio), was dialyzed overnight against 2 L buffer A at 4°C. The dialyzed sample was reloaded on a 5 mL TALON column to retain TEV protease and MBP proteins. The flow-through was collected and dialyzed against 5 L buffer B overnight. The eluted *Dp*IseK ($\varepsilon280 = 35{,}410\ M^{-1}cm^{-1}$) was concentrated by ultrafiltration (Sartorius VIVASPIN TURBO 15 (30,000MWCO)) and used for ITC assays.

The recombinant *Bw*TauF plasmid was transformed into BL21 (DE3) cells. The transformant was grown in LB medium containing kanamycin (50 µg/mL) at 37°C in flasks in a shaker incubator at 220 rpm, and induced for the expression of TauF with 0.4 mM IPTG for overnight at 18°C. The cells were harvested by centrifugation at 8000 g for 10 min.

The harvested cells (4 g) were suspended in 25 mL of lysis buffer [50 mM Tris-HCl (pH 8.0), 1 mM PMSF, 0.2 mg/mL lysozyme, 0.03% Triton-X, and 25 µg/mL DNaseI]. The cell suspension was frozen at −80°C, and then thawed and incubated at room temperature for 40 min to allow lysis. The lysate was subjected to 1% streptomycin sulfate precipitation to remove nucleic acids, and the precipitated DNA was removed by centrifugation (20000 g for 10 min at 4°C). Protein solution was then applied to a 5 mL TALON resin (GE), pre-equilibrated with buffer B. The protein was eluted with elution buffer containing 150 mM imidazole. The eluted protein was precipitated with solid $(NH_4)_2SO_4$ to 70% saturation and isolated by centrifugation (20000 g for 5 min at 4°C). The pellet was dissolved in 2.5 mL of buffer containing 20 mM Tris/HCl, pH 7.5, 100 mM KCl, and 1 mM DTT, and desalted using a Sephadex G-25 column. The eluted protein was frozen in aliquots in liquid N_2, and stored at −80°C. The purified *Bw*TauF was examined by SDS-PAGE on a 10% gel.

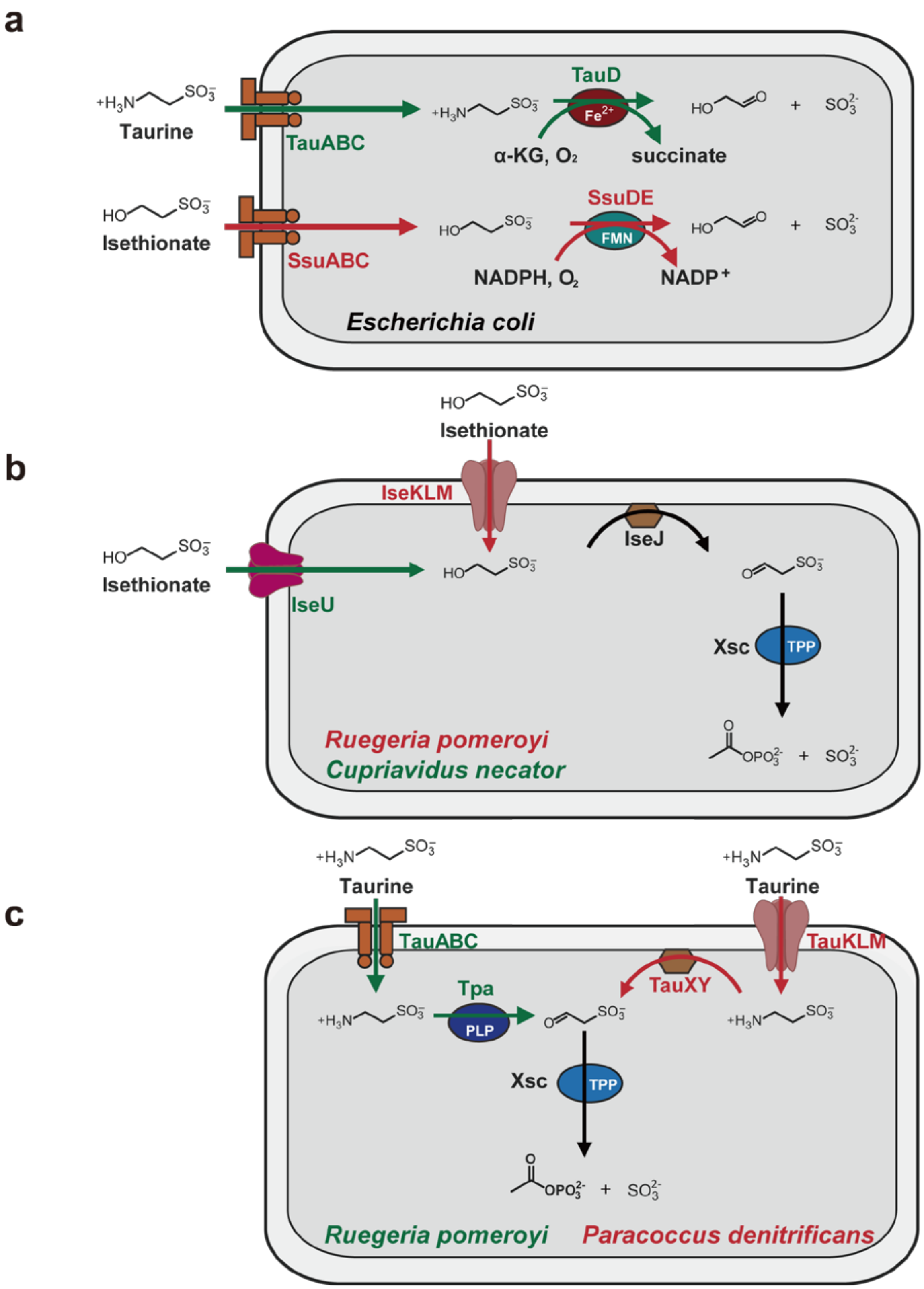

Supplementary Fig. 1 | Known pathways for bacterial taurine and isethionate degradation. a, O_2-dependent pathways for sulfur assimilation from taurine and isethionate in *E. coli*[2]. **b,** Isethionate dissimilation pathways in environmental bacteria[3]. **c,** Taurine dissimilation pathways in environmental bacteria[4]. TauABC – taurine ABC transporter; SsuABC – aliphatic sulfonate ABC transporter; TauD – α-ketoglutarate-dependent taurine dioxygenase; SsuDE – alkanesulfonate

monooxygenase; IseKLM – isethionate TRAP transporter; IseU – isethionate MFS transporter; IseJ – isethionate dehydrogenase; Xsc – sulfoacetaldehyde acetyltransferase; Tpa – taurine pyruvate aminotransferase; TauKLM – taurine TRAP transporter; TauXY – taurine dehydrogenase.

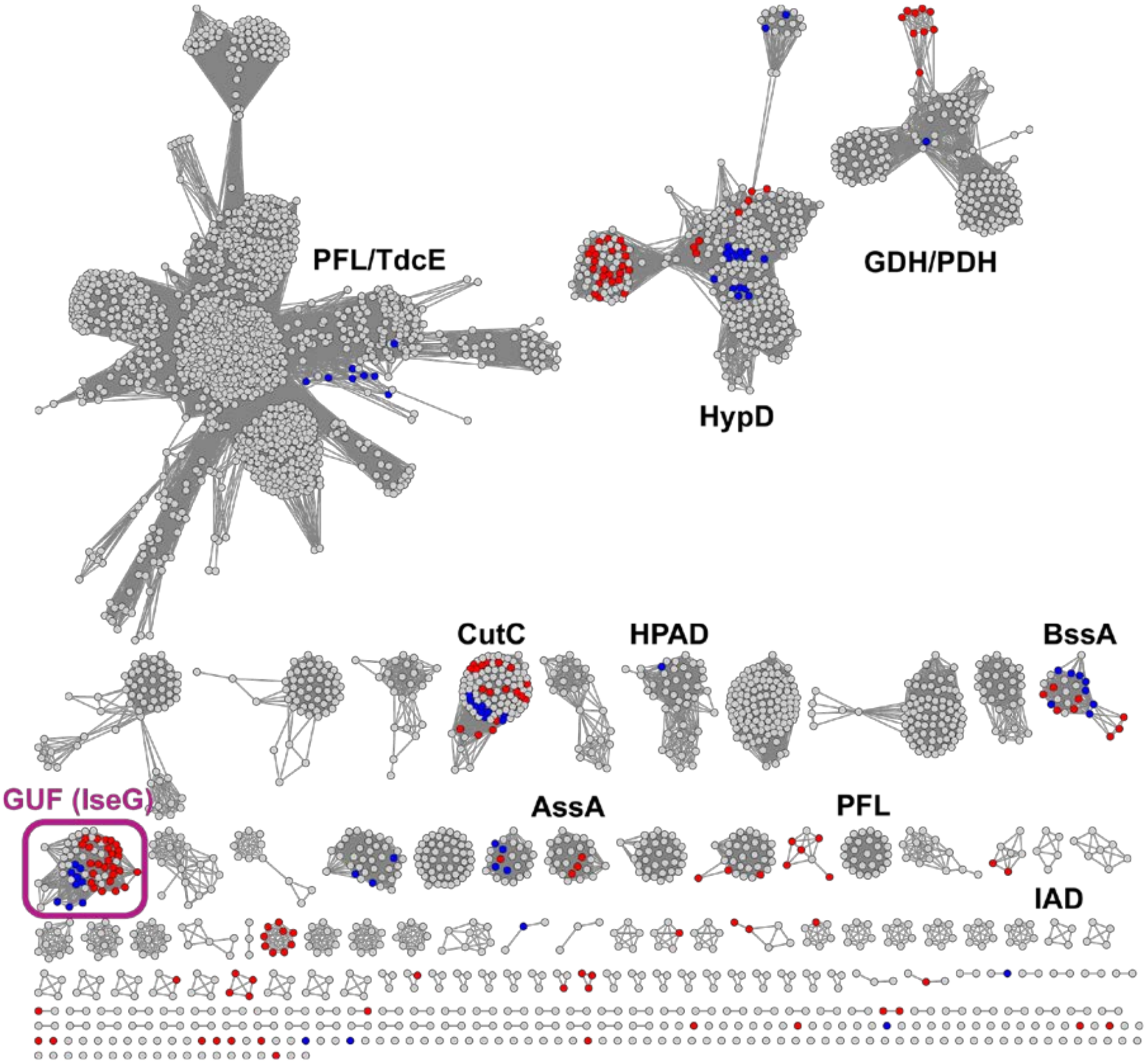

Supplementary Fig. 2 | SSN for the GRE superfamily (IPR004184) displayed at the E-value cut-off of 10^{-299}. The most highly related proteins are grouped together in clusters, putatively sharing the same function. Each node represents a group of proteins sharing more than 90% sequence identity. Clusters containing enzymes with known functions are labelled. The known enzymes and their UniProt accession IDs are: PFL - pyruvate formate lyase (P09373, O32797); TdcE - 2-keto acid formate-lyase (P42632); CutC - choline-trimethylamine lyase (Q30W70, A0A0M3KL44); PDH - propanediol dehydratase (Q1A666); GDH - glycerol dehydratase (Q8GEZ8); HypD - *trans*-4-hydroxy-$_{\text{L}}$-proline dehydratase (A0A125YDI6); BssA - benzylsuccinate synthase (O87943); AssA - alkylsuccinate synthase (B8FEM4); HPAD - *p*-hydroxyphenylacetate decarboxylase (Q18CP5, A0A0E3JS98, A0A1D3UC78); IAD – indoleacetate decarboxylase (A0A100YXA1); and IseG – isethionate lyase (Q727N1), reported in this study. Nodes coloured red contain proteins from gram negative SSRB in the class Deltaproteobacteria (orders Desulfobacterales, Desulfovibrionales, Desulfurellales and Desulfuromonadales), and nodes coloured blue contain proteins from gram positive SSRB in the family Peptococcaceae (genus *Desulfitibacter*, *Desulfitobacterium*, *Desulfocucumis*, *Desulfonispora*, *Desulfosporosinus* and *Desulfotomaculum*), showing the prevalence of phylogenetically diverse SSRB in the IseG cluster. Source data are provided as Supplementary Data 1.

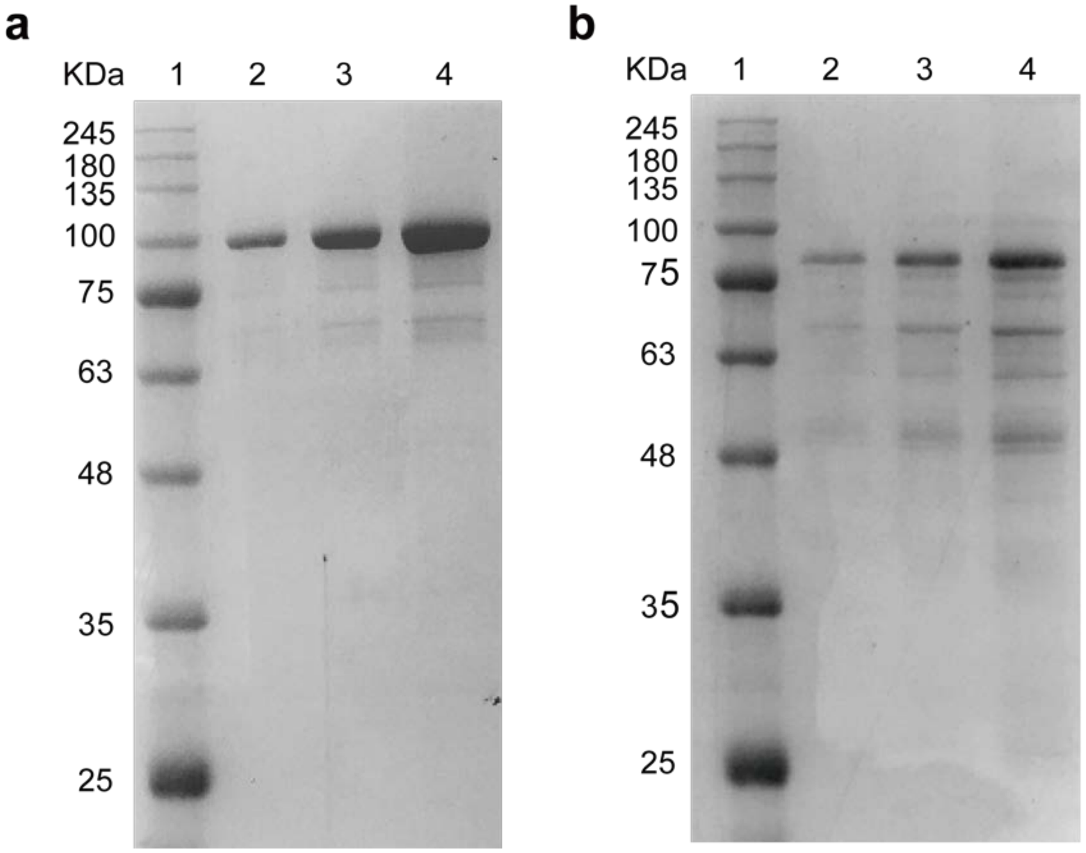

Supplementary Fig. 3 | SDS-PAGE analyses of purified IseG and MBP-IseH used in enzymatic assays and biochemical characterization. a, IseG. **b,** MPB-IseH. 10% SDS gel with: lane 1, protein molecular weight marker; and lane 2-4: 1, 2, 4 µg of IseG or MBP-IseH. Source data are provided as a Source Data file.

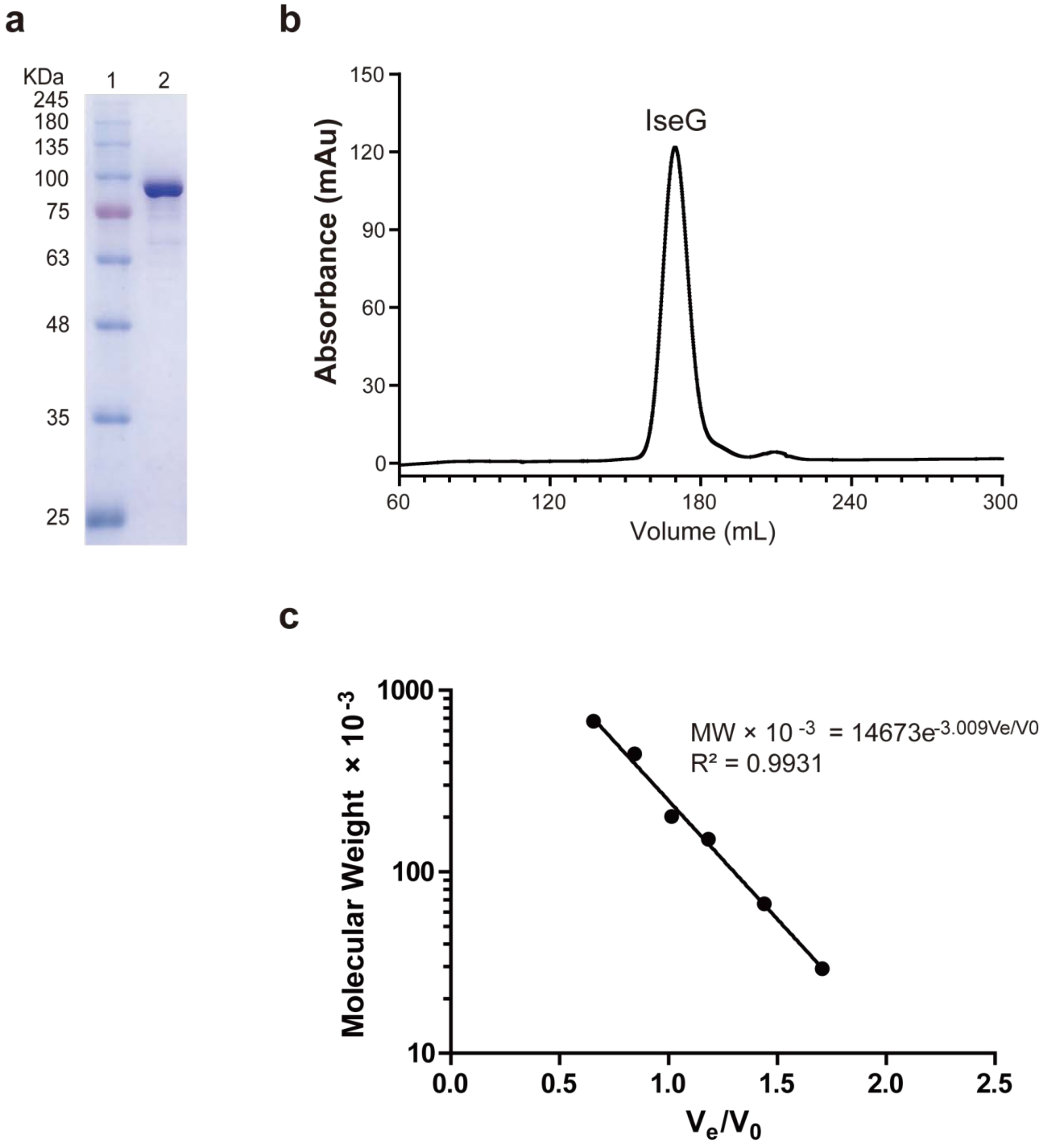

Supplementary Fig. 4 | SDS-PAGE and SEC analyses of purified IseG (-23 a.a.) used for protein crystallization. a, 10% SDS gel with: lane 1, protein molecular weight marker; and lane 2, 2 µg of purified IseG (-23 a.a.). **b,** Elution profile of IseG (-23 a.a.) using Superdex 200 gel filtration chromatography to determine IseG molecular weight, estimated to be 196 kDa. **c,** Calibration plot based on the molecular weight standards, including bovine thyroglobulin (669kDa), horse apoferritin (443 kDa), sweet potato β-Amylase (200 kDa), yeast alcohol dehydrogenase (150 kDa), BSA (66 kDa), and bovine carbonic anhydrase (29 kDa) (Sigma MWGF 1000-1KT). Source data are provided as a Source Data file.

a

```
DvIseH      MSSIADRKTTGITFNIQKYSVHDGPGIRTVVFLKGCPLKCRWCSNPESQRKSVELAYNTG   60
CsHPADAE    ------MKEKGLIFDIQSFSVHDGPGCRTSVFFIGCPLQCKWCANPESWTKKKHIMVAEN   54
CdHPADAE    --MSSQKQLEGMIFDVQSFSVHDGPGCRTTVFLNGCPLSCKWCANPESWTVRPHMMFSEL   58
TaBssD      -------MKIPLITEIQRFSLQDGPGIRTTIFLKGCPLRCPWCHNPETQDARQEFYFYPD   53
CbGDHAE     ----MSKEIKGVLFNIQKFSLHDGPGIRTIVFFKGCSMSCLWCSNPESQDIKPQVMFNKN   56
DaCutD      -----MIERKALIFNIQKYNMYDGPGVRTLVFFKGCPLRCKWCSNPEGQLRQYQVLYKEN   55
CdHypD      --------MNPLVINLQKCSIHDGPGIRSTVFFKGCPLECVWCHNPESQTYTKQVLYNEE   52
EcPflA      ------MSVIGRIHSFESCGTVDGPGIRFITFFQGCLMRCLYCHNRDTWDT---------   45
RiPDAE      --MKEYLNTSGRIFDIQRYSIHDGPGVRTIVFLKGCALRCRWCCNPESQSFEVETMTING   58
EcNrdG      ----------MNYHQYYPVDIVNGPGTRCTLFVSGCVHECPGCYNKSTWRVNSGQPFTK-   49
1CLF        ------------------------------------------------------AYKIAD   6
1DUR        ------------------------------------------------------AYVIND   6
2ZVS        ------------------------------------------------------ALLITK   6
1RGV        ------------------------------------------------------ALYIND   6
1BWE        ------------------------------------------------------AYVITE   6

DvIseH      RCLTLAKCVRCVEICTAGAIS--RAEDDT--ISIDRALCNDCE----Q-LCSGACPSNAL   111
CsHPADAE    VCKWKNGCRSCINACSHDSIK--FSEDGK--LKISWDTCEKCE----TFDCVNMCPNNAL   106
CdHPADAE    SCQYENGCTVCHGKCKNGALS--FNLDNK--PVIDWNICKDCE----SFECVNSCYYNAF   110
TaBssD      RCV---GCGRCVAVCPAETSRLVRNSDGRTIVQIDRTNCQRCM------RCVAACLTEAR   104
CbGDHAE     LCT---KCGRCKSQCKSAAID--M-NSEYR---IDKSKCTECT------KCVDNCLSGAL   101
DaCutD      LCV---HCGACVPVCPAGVHT--ISASTLRHGFAEGAQCIGCR------RCEDVCPSSAL   104
CdHypD      RCS---KCEACINICPHKAIY--KGETKIC---LDQDKCEFCE------TCLDYCVNNAR   98
EcPflA      ------------------------------------------------------------   45
RiPDAE      KP----------------------------------------------------------   60
EcNrdG      ------------------------------------------------------------   49
1CLF        SCV---SCGACASECPVNAIS--QGDSI---FVIDADTCIDCG------NCANVCPVGAP   52
1DUR        SCI---ACGACKPECPVNCIQ--EG-SI---YAIDADSCIDCG------SCASVCPVGAP   51
2ZVS        KCI---NCDMCEPECPNEAIS--MGDHI---YEINSDKCTECVGHYETPTCQKVCPIPNT   58
1RGV        DCT---ACDACVEECPNEAIT--PGDPI---YVIDPTKCSECVGAFDEPQCRLVCPADC-   57
1BWE        PCI-GTKCASCVEVCPVDCIH--EGEDQ---YYIDPDVCIDCG------ACEAVCPVSAI   54
```

b

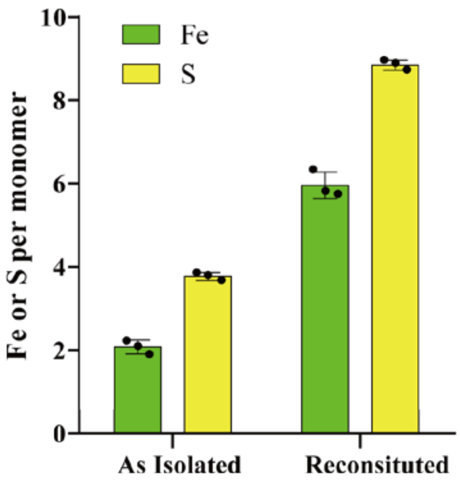

Supplementary Fig. 5 | [Fe-S] cluster quantitation in IseH. a, Multiple sequence alignment of N-terminal regions of IseH with previously studied GRE activating enzymes and structurally characterized 2-[4Fe-4S] ferredoxins, modified from Selvaraj *et al*.[5] The three cysteine residues that coordinate the radical SAM [4Fe-4S] cluster [6], present in all the sequences, are coloured blue. For sequences containing an additional ferredoxin-like domain [5], the eight cysteine residues thought to coordinate the two auxiliary [4Fe-4S] clusters in that domain are coloured red. The sequences are for the activating enzymes of: DvIseH (UniProt ID: Q727N0), CsHPADAE (Q38HX2) – *Clostridium scatologenes* HPAD, CdHPADAE (Q84F14) – *Clostridium difficile* HPAD, TaBssD (O87941) – *Thauera aromatic* benzylsuccinate synthase, CbGDHAE (Q8GEZ7) – *Clostridium butyricum* glycerol dehydratase, DaCutD (Q30W71) – *Desulfovibrio alaskensis* choline-trimethylamine lyase, CdHypDAE (A0A069AMK2) – *Clostridium difficile* 4-hydroxyproline dehydratase, EcPflA (P0A9N4) – *Escherichia coli* pyruvate formate lyase, RiPDHAE (Q1A665) – *Roseburia inulinivorans* 1,2-propanediol dehydratase, EcNrdG (P0A9N8) – *Escherichia coli* anaerobic ribonucleotide reductase. 1CLF, 1DUR, 2ZVS, 1RGV, 1BWE are the PDB accessions of structurally characterized ferredoxins. **b,** Fe and S contents of IseH. The assays were performed in triplicate and are presented with standard deviations. Green bars represent Fe contents, yellow bars represent S contents. Source data are provided as a Source Data file.

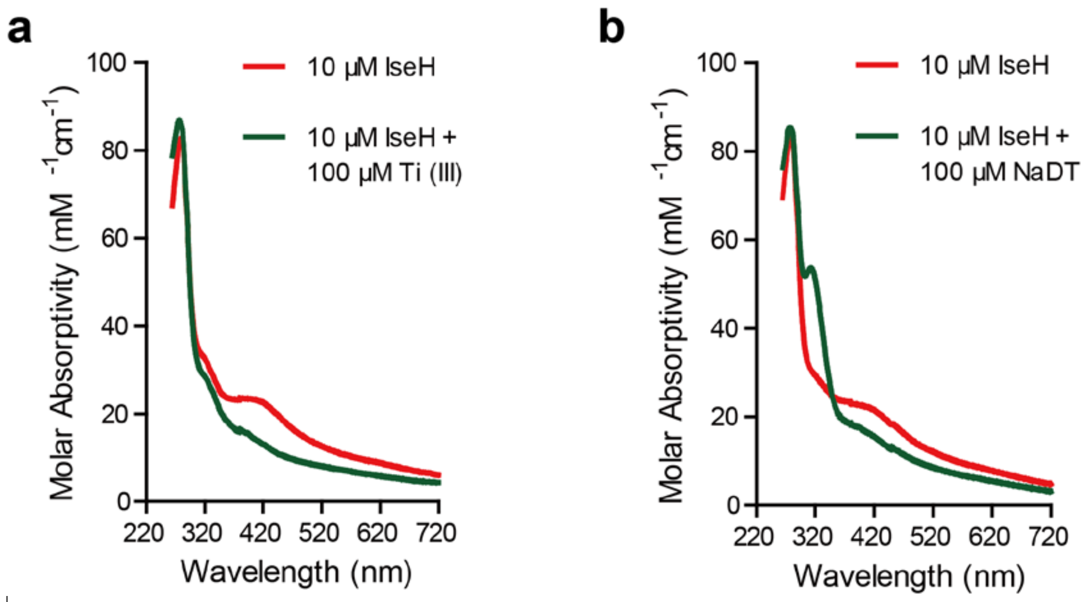

Supplementary Fig. 6 | UV-Vis absorption spectra of the reconstituted IseH. Like other [4Fe-4S]-containing proteins, the feature at 420 nm corresponding to $[4Fe\text{-}4S]^{2+}$ clusters in IseH disappears upon reduction with strong reductants: **a,** Ti(III) citrate or **b,** sodium dithionite (NaDT). Source data are provided as a Source Data file.

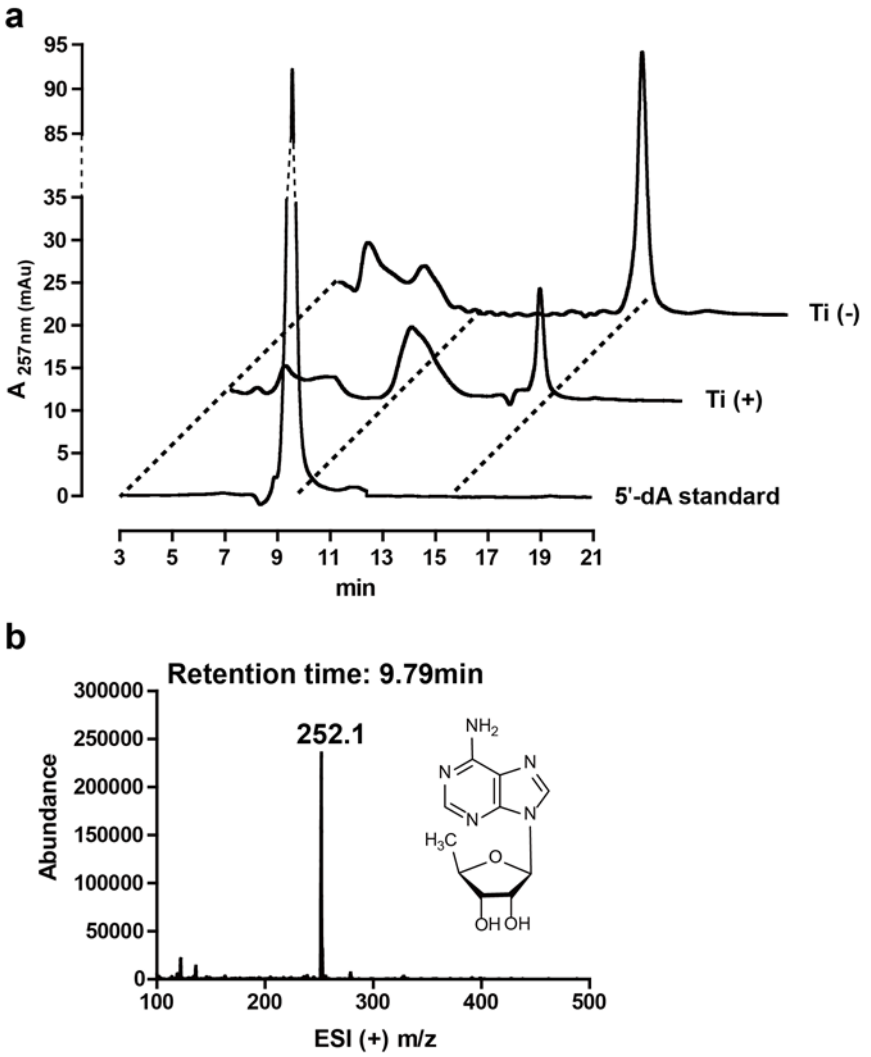

Supplementary Fig. 7 | LC-MS analysis of 5'-deoxyadenosine (5'-dA) formed upon incubation of IseH with SAM and the strong reductant Ti(III) citrate. a, Elution profiles of the assays in the presence and absence of Ti(III), and the commercial 5'-dA standard. **b,** Positive ionization mass spectrum of the 5'-dA peak in the assay eluted at 9.79 min. Source data are provided as a Source Data file.

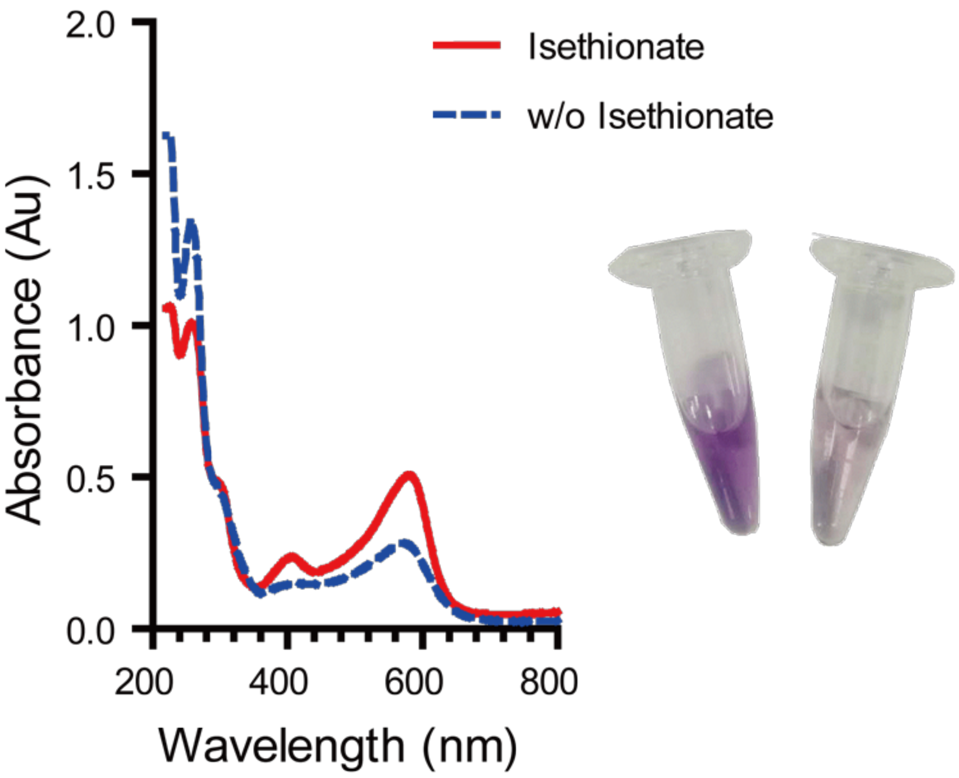

Supplementary Fig. 8 | Detection of sulfite formation in the IseG-catalysed isethionate cleavage by a colorimetric Fuchsin assay. UV-Vis absorption spectra of the complete assay and the negative control omitting isethionate are shown in red and blue, respectively. The background signal (blue) is the result of a reaction of the Fuchsin-formaldehyde reagent with sulfide and thiols present in the proteins and buffer [7]. Photograph: Fuchsin assay reaction mixture of the complete assay (left) and the negative control with substrate isethionate omitted (right) in Eppendorf tubes. Source data are provided as a Source Data file.

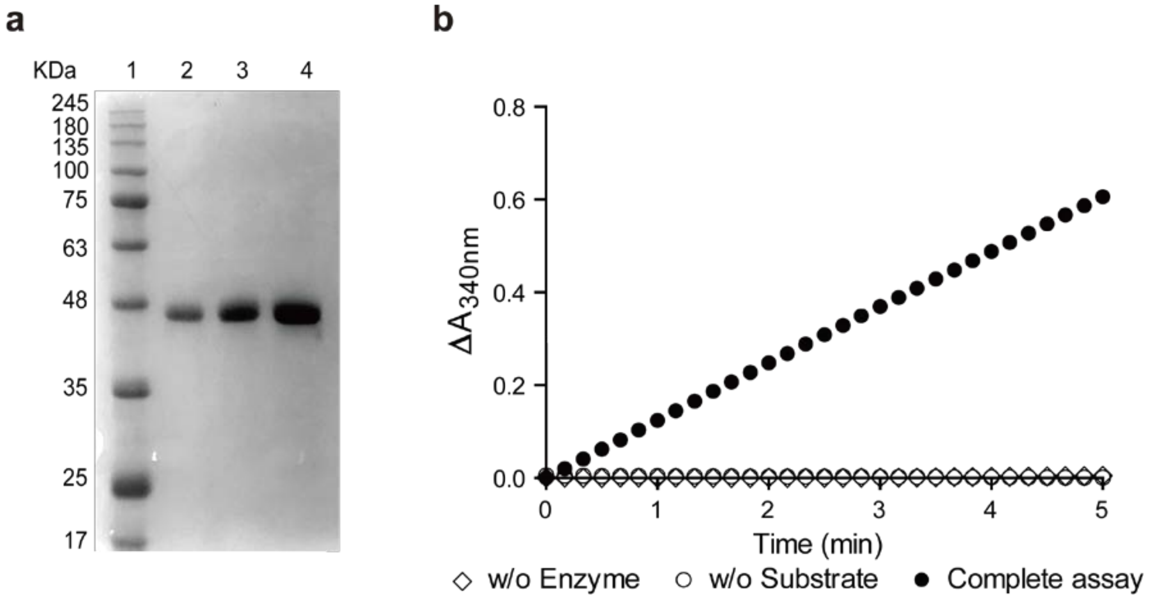

Supplementary Fig. 9 | SDS-PAGE gel and enzyme activity analyses of purified *Sc*ADH1 used as a coupling enzyme in IseG assays. a, 12% SDS gel of purified *Sc*ADH1 with: lane1, molecular weight marker; lane 2-4, 1, 2, 4 µg of *Sc*ADH1. **b,** Enzyme activity assay monitoring NADH formation accompanying ethanol oxidation by *Sc*ADH1. Source data are provided as a Source Data file.

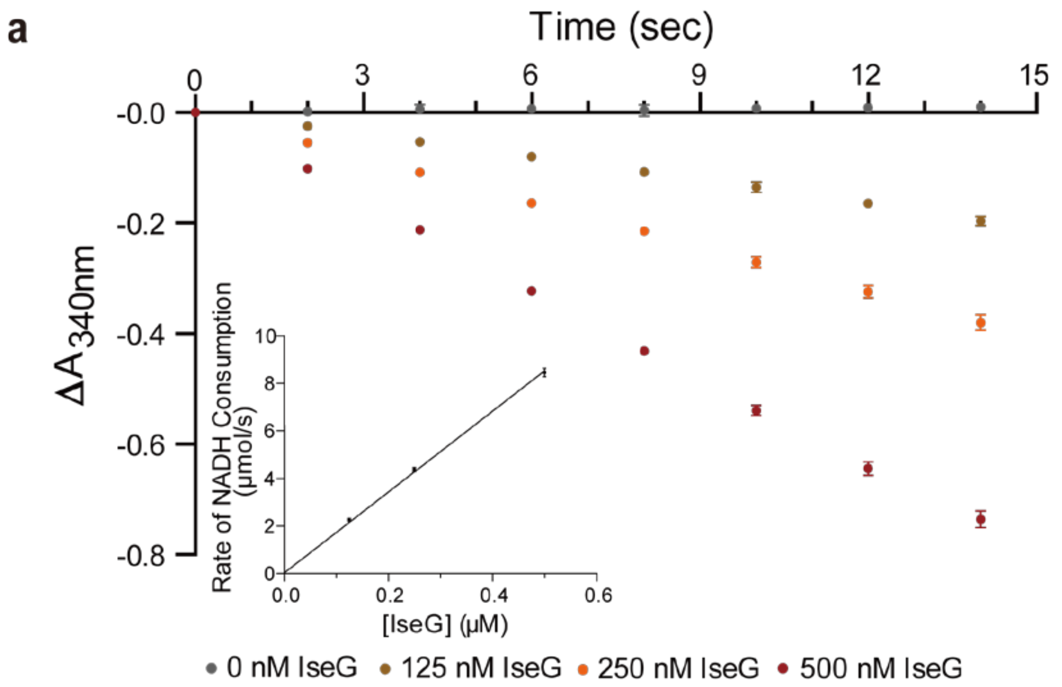

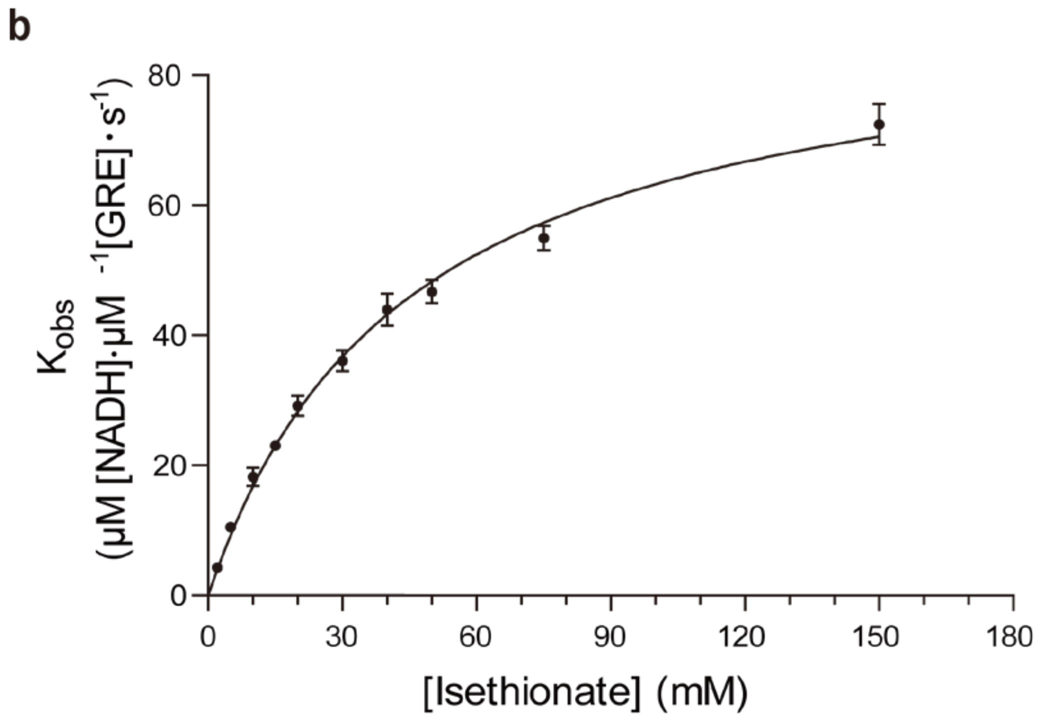

Supplementary Fig. 10 | IseG enzyme kinetics monitored by NADH loss at 340 nm. a, Dose-dependent enzyme activities. IseG enzyme activity was measured using an ADH coupled assay. 0, 125, 250, 500 nM of IseG (grey, brown, orange and red, respectively) were assayed for activity with 400 mM isethionate, 10 µM *Sc*ADH1 and 0.2 mM NADH. **b,** Michaelis–Menten kinetics of IseG. 250 nM IseG was assayed for activity in a coupled assay with 10 µM *Sc*ADH1 and varying isethionate concentrations. Error bars represent the standard deviation of three individual experiments. Source data are provided as a Source Data file.

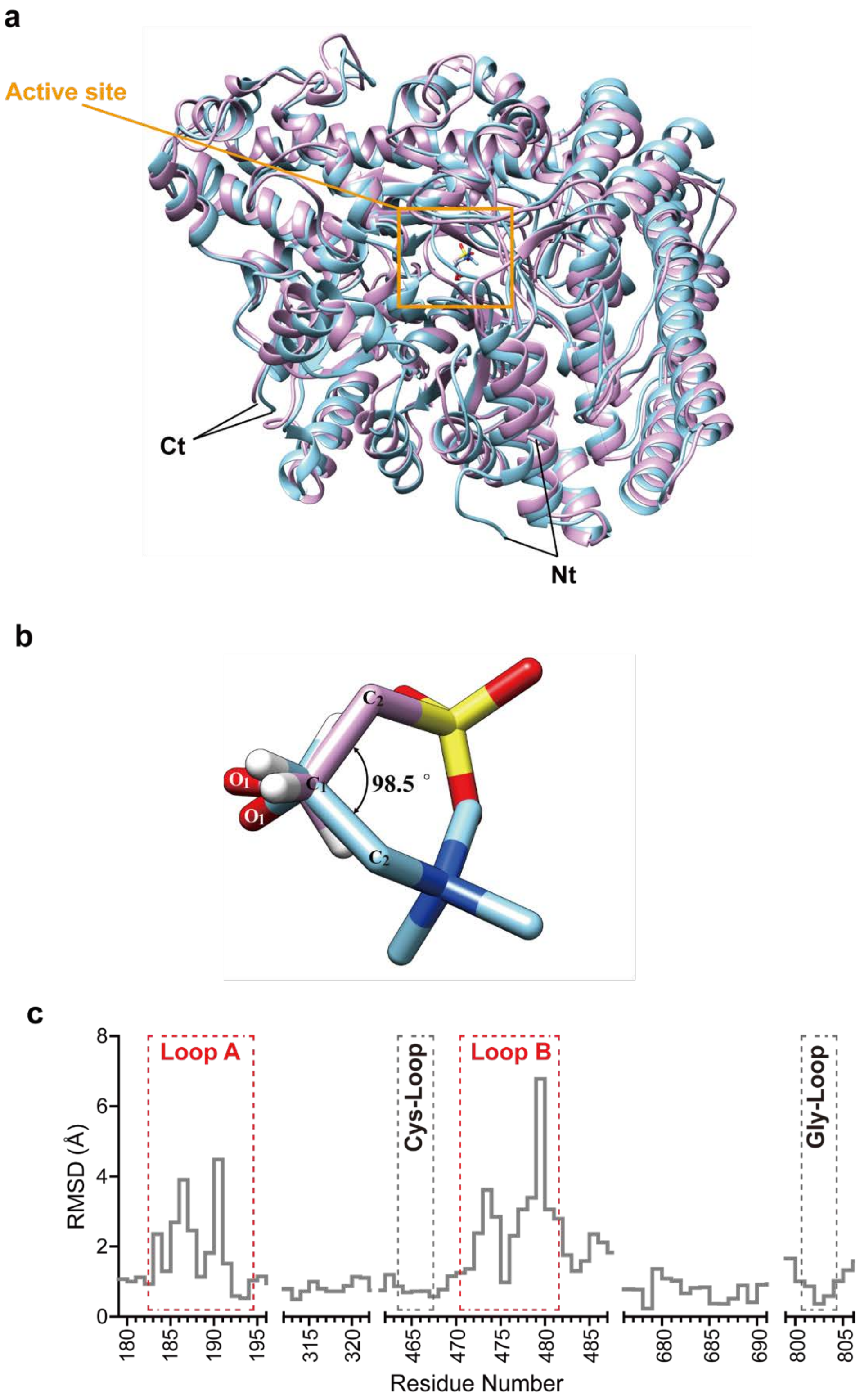

Supplementary Fig. 11 | Comparison of IseG and CutC crystal structures. a, Superposition of IseG crystal structure (plum) with CutC (cyan, PDB code 5FAU). The active sites are indicated by an orange box. N-terminal and C-terminal ends of two proteins are labelled. **b,** Comparison of the conformations of the substrates isethionate (plum) and choline (cyan). The angle between the C1-C2 axes of choline and isethionate is labelled. The C1 hydrogens of both isethionate and choline are shown. **c,** Plot showing the root mean square deviation (RMSD) between corresponding residues in the IseG and CutC active sites. Four critical loops are highlighted by the boxes.

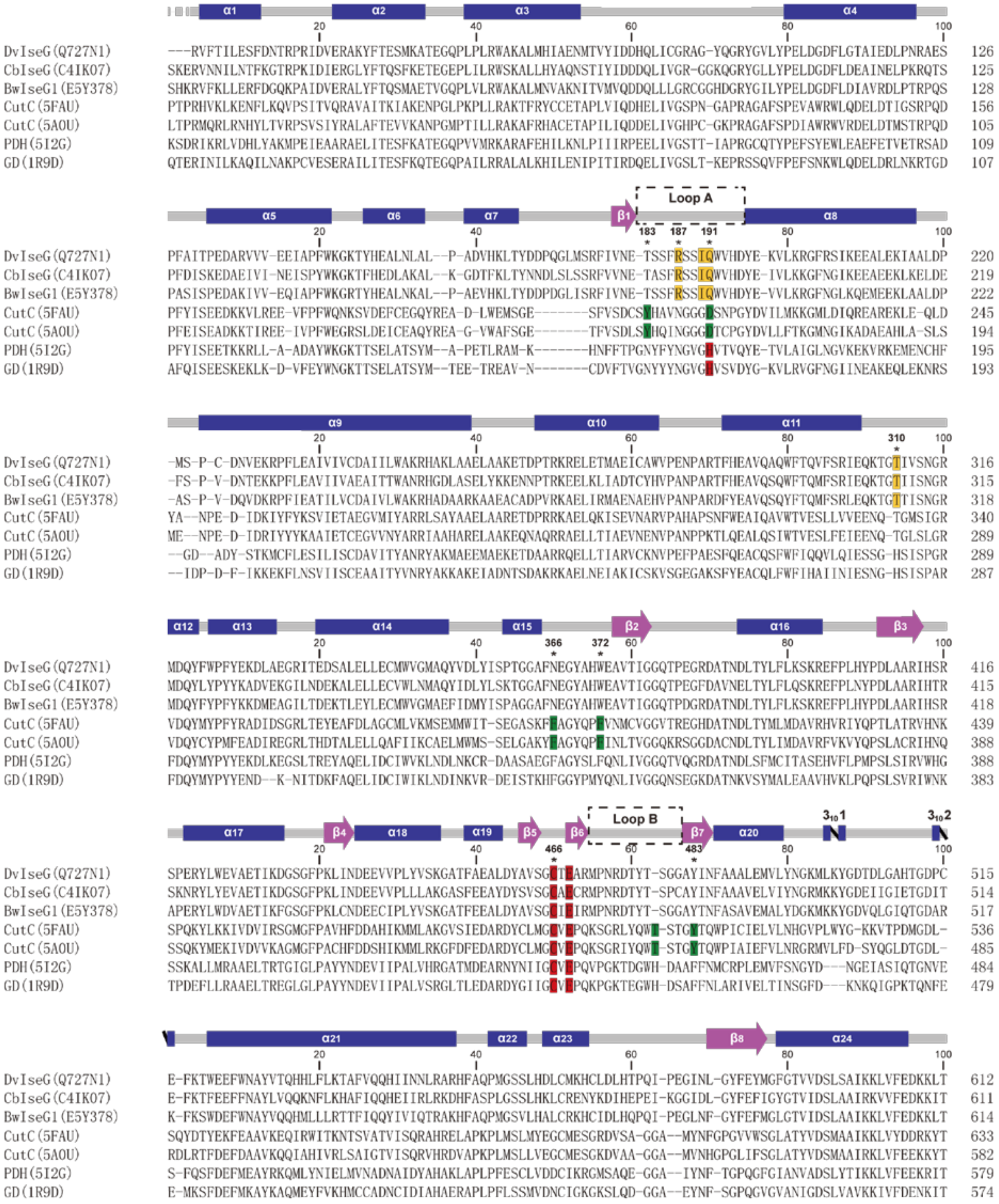

α1 α2 α3 α4
α5 α6 α7 β1 Loop A α8
183 187 191
α9 α10 α11
310
α12 α13 α14 α15 β2 α16 β3
366 372
α17 β4 α18 α19 β5 β6 Loop B β7 α20 3₁₀1 3₁₀2
466 483
α21 α22 α23 β8 α24
DvIseG(Q727N1)
CbIseG(C4IK07)
BwIseG1(E5Y378)
CutC(5FAU)
CutC(5A0U)
PDH(5I2G)
GD(1R9D)

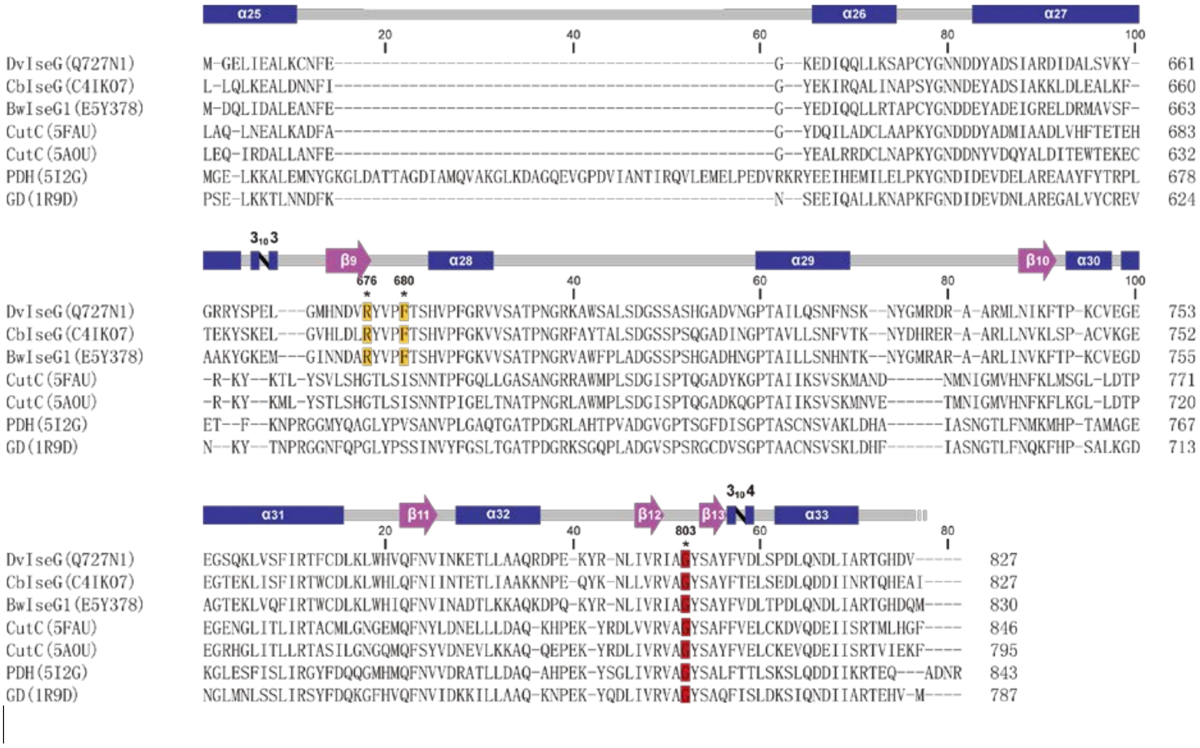

Supplementary Fig. 12 | Structure-based multiple sequence alignments of IseGs with selected previously characterized GREs. The Gly and Cys residues involved in radical chemistry, as well as the Glu residue involved in the C1-OH deprotonation and the His residue involved in C2-OH protonation (required for PDH and GDH) are highlighted in red. Residues in the active site of CutC that interact with the substrate trimethylamino group are highlighted in green. Residues in the active site of our IseG crystal structure that interact with the substrate sulfonate group are highlighted in yellow.

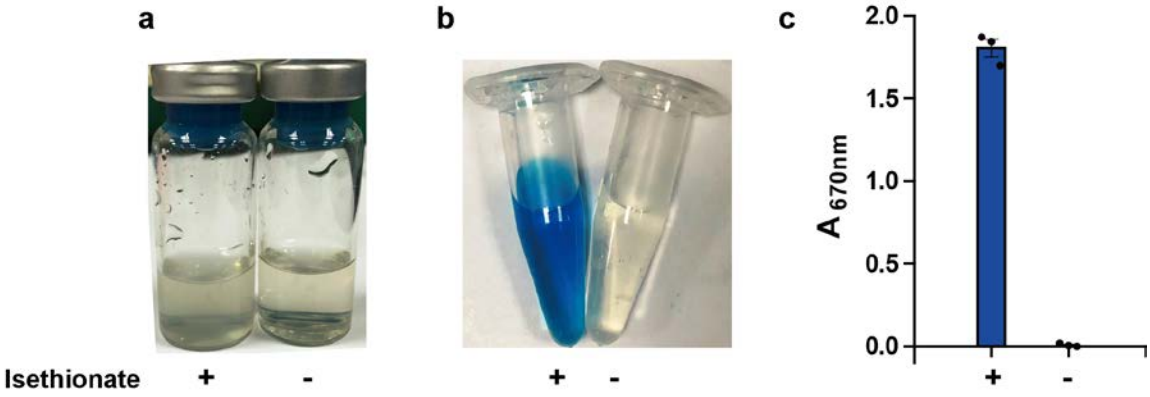

Supplementary Fig. 13 | Isethionate-dependent growth of *D. piger* and H_2S formation. a, Anaerobic cell growth of *D. piger* is dependent on the presence of isethionate. **b,** Formation of methylene blue by reacting the headspace gas with DPD. **c,** Absorbance of the respective reaction mixtures at 670 nm. The assays were performed in triplicate and are presented with standard deviations. Source data are provided as a Source Data file.

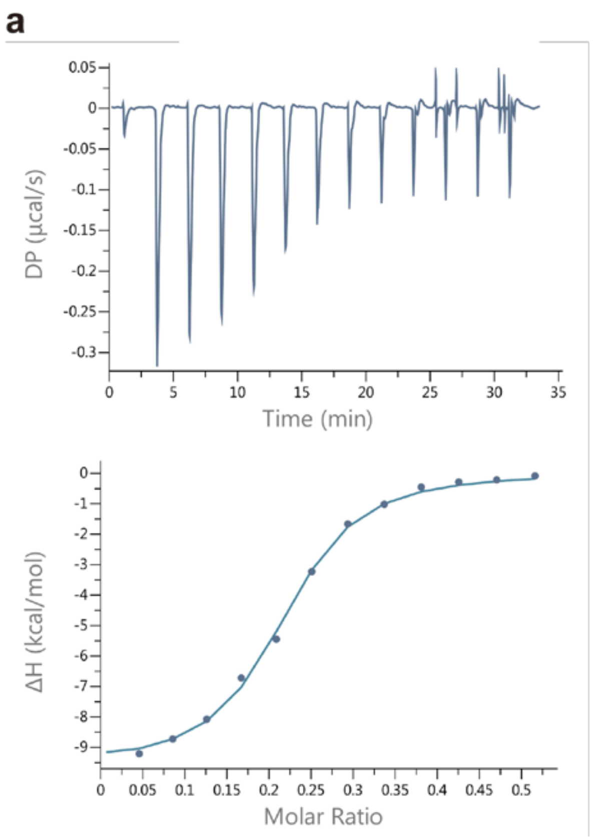

Supplementary Fig. 14 | ITC binding isotherms show the interaction between *Dp*IseK and isethionate. a, titration of isethionate. Bottom solid line represents the fit. The fitted K_d is 0.5 µM; ΔH is -9.64 kcal/mol; -TΔS is 1.03 kcal/mol. **b,** titration of taurine. Source data are provided as a Source Data file.

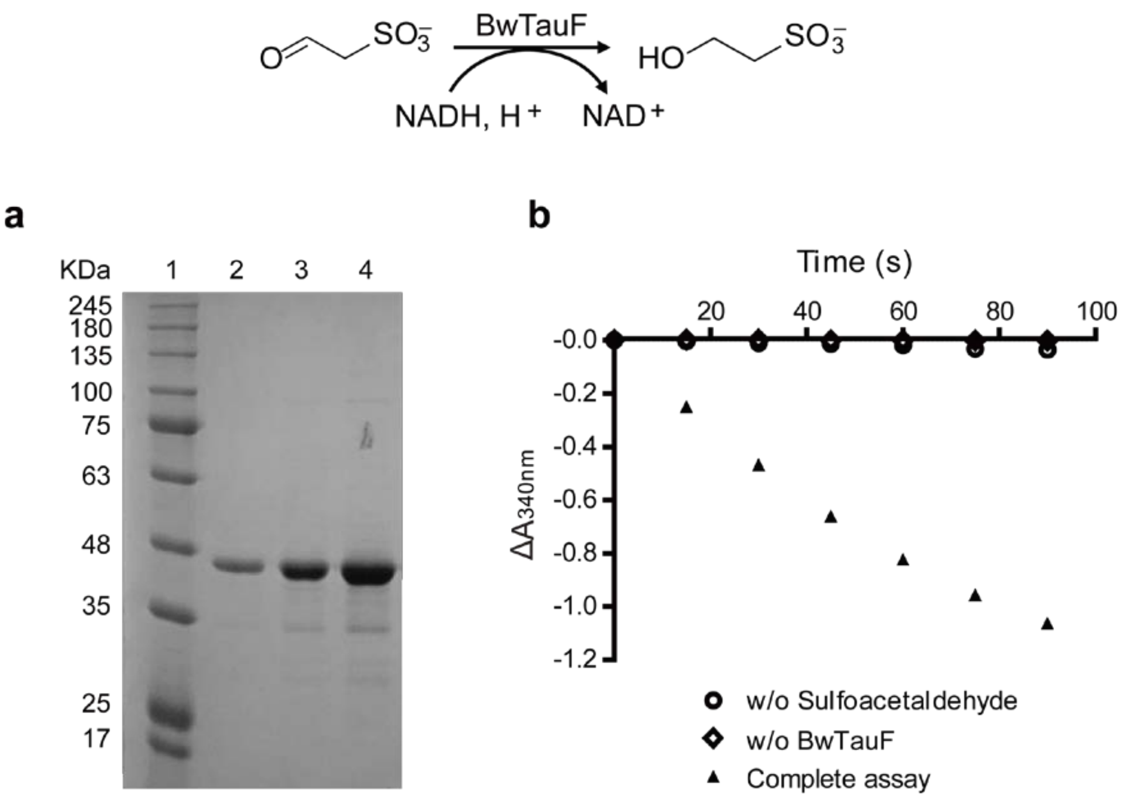

Supplementary Fig. 15 | SDS-PAGE gel and enzyme activity analyses of purified *Bw*TauF, as shown in the reaction equation. a, 10% SDS gel with: lane 1, molecular weight marker; and lane 2-4, 1, 2, 4 µg of *Bw*TauF. **b,** The assays monitor NADH consumption accompanying sulfoacetaldehyde reduction by *Bw*TauF. Source data are provided as a Source Data file.

Supplementary Table 1 | Correlation between ability to dissimilate C2 sulfonates and presence of putative IseG or putative Xsc (within the InterPro family IPR017820) in the genome of various sequenced strains of SSRB.

SSRB strain	Xsc	IseG	Taurine	Isethionate	Ref.	Genome sequenced
Desulfonispora thiosulfatigenes DSM 11270	A0A1W1VSZ3	X	✓	X	[8,9]	FWWT01000000
Desulforhopalus singaporensis DSM 12130	A0A1H0JWS8	X	✓	X	[10]	FNJI01000000
Desulfotalea psychrophila DSM 12343	Q6APX2	Q6AIS4	✓*	?	[11]	CR522870
Desulfobacter postgatei DSM 2034	X	X	X	X	[12]	AGJR02000000
Desulfobacterium autotrophicum DSM 3382	X	X	X	X	[12]	CP001087
Desulfobulbus propionicus DSM 2032	X	X	X	X	[12]	GCA_000186885
Desulfuromonas acetoxidans DSM 684	X	X	X	X	[12]	AAEW02000000
Desulfomicrobium norvegicum DSM 1741	A0A1I4MM43	A0A1I4JGT9	X	✓	[12]	FOTO00000000
Desulfitobacterium chlororespirans DSM 11544	A0A1M7UVY6, A0A1M7UXG6	X	X	X	[13]	FRDN01000000
Desulfitobacterium dehalogenans DSM 9161	I4A5M2, I4AE53	I4A4E8	X	✓	[13]	CP003348
Desulfitobacterium hafniense DCB-2 DSM 10664	B8FXN4, B8FZH4	B8G1R0	X	✓	[13]	CP001336
Desulfovibrio piger DSM 749	X	B6WXM2	X	✓	This study	ABXU01000000
Bilophila wadsworthia RZATAU	X	E5Y378, (*Bilophila Wadsworthia* 3_1_6)*	✓	✓	[14,15], this study	ADCP02000000 (*Bilophila Wadsworthia* 3_1_6)*

The genome of *Bilophila wadsworthia* RZATAU is not sequenced but based on 16S rDNA, it is closely related to the sequenced strain *B. wadsworthia* 3_1_6.

Supplementary Table 2 | Oligonucleotides used for cloning and sequencing.

Oligos	Target	Sequence (5' to 3')	Application
1F	IseG	(5'-TACTTCCAATCCAATGCACGTTACCGCGC CACACATGA-3')	Construct HMT-IseG(-23aa)
1R	IseG	(5'-TTATCCACTTCCAATGTTATTACATAACA TCATGACCGGTGCGT-3')	Construct HMT-IseG(-23aa)
2F	IseG	(5'-TTTGCCATTACCCCGGCCGCCGCCGCCGT GGTTGTGGAAGAAATT-3')	QuickChange mutagenesis
2R	IseG	(5'-AATTTCTTCCACAACCACGGCGGCGGCGG CCGGGGTAATGGCAAA-3')	QuickChange mutagenesis
3F	*Sc*ADH1	(5'-GCCAGAGCGGATCAGGATCTATCCCAGA AACTCAAAAAGGTG-3')	Construct pET-28a(+)-His_6-*Sc*ADH1
3R	*Sc*ADH1	(5'-CCAATTGAGATCTGCCATATGTTATTTAG AAGTGTCAACAACGTATC-3')	Construct pET-28a(+)-His_6-*Sc*ADH1

Supplementary Table 3 | Data collection and refinement statistics for the IseG crystal.

**Data collection**	
Space group	*P 1 21 1*
**Cell dimension (Å)**	
a, b, c (Å)	110.1, 159.4, 115.4
α, β, γ, (°)	90.0, 91.7, 90.0
Resolution (Å)	48.13-2.40 (2.48- 2.40)
R_{sym} or R_{merge}	0.146 (0.559)
I/σ(I)	6.9 (1.7)
Completeness (%)	98.40 (93.38)
Redundancy	3.2 (2.6)
**Refinement**	
Resolution (Å)	48.13-2.40
No. of reflections	153165
R_{work}/R_{free}	0.2116/0.2620
**No. of atoms**	
Protein	24931
Ligand/ion	64
Water	737
**B-factors**	
Protein	27.25
Ligand/ion	29.26
Water	24.86
**R.M.S. deviations**	
RMSD length (Å)	0.008
RMSD angle (°)	0.9

One crystal was used
*Highest resolution shell is shown in parenthesis.

Supplementary References

1 Sievers, F. *et al.* Fast, scalable generation of high-quality protein multiple sequence alignments using Clustal Omega. *Mol Syst Biol* **7** (2011).

2 van der Ploeg, J. R., Eichhorn, E. & Leisinger, T. Sulfonate-sulfur metabolism and its regulation in Escherichia coli. *Arch Microbiol* **176**, 1-8 (2001).

3 Weinitschke, S., Sharma, P. I., Stingl, U., Cook, A. M. & Smits, T. H. Gene clusters involved in isethionate degradation by terrestrial and marine bacteria. *Applied and environmental microbiology* **76**, 618-621 (2010).

4 Cook, A. M. & Denger, K. Metabolism of taurine in microorganisms: a primer in molecular biodiversity? *Adv Exp Med Biol* **583**, 3-13 (2006).

5 Selvaraj, B., Buckel, W., Golding, B. T., Ullmann, G. M. & Martins, B. M. Structure and Function of 4-Hydroxyphenylacetate Decarboxylase and Its Cognate Activating Enzyme. *J Mol Microbiol Biotechnol* **26**, 76-91 (2016).

6 Vey, J. L. *et al.* Structural basis for glycyl radical formation by pyruvate formate-lyase activating enzyme. *Proc Natl Acad Sci U S A* **105**, 16137-16141 (2008).

7 Steigmann, A. Acid-Bleached Fuchsin Solution as Analytical Reagent. *Analytical Chemistry* **22**, 492-493 (1950).

8 Denger, K., Stackebrandt, E. & Cook, A. M. Desulfonispora thiosulfatigenes gen. nov., sp. nov., a taurine-fermenting, thiosulfate-producing anaerobic bacterium. *Int J Syst Bacteriol* **49 Pt 4**, 1599-1603 (1999).

9 Denger, K., Ruff, J., Rein, U. & Cook, A. M. Sulphoacetaldehyde sulpho-lyase (EC 4.4.1.12) from Desulfonispora thiosulfatigenes: purification, properties and primary sequence. *Biochem J* **357**, 581-586 (2001).

10 Lie, T. J., Clawson, M. L., Godchaux, W. & Leadbetter, E. R. Sulfidogenesis from 2-aminoethanesulfonate (taurine) fermentation by a morphologically unusual sulfate-reducing bacterium, Desulforhopalus singaporensis sp. nov. *Appl Environ Microbiol* **65**, 3328-3334 (1999).

11 Denger, K., Smits, T. H. & Cook, A. M. Genome-enabled analysis of the utilization of taurine as sole source of carbon or of nitrogen by Rhodobacter sphaeroides 2.4.1. *Microbiology* **152**, 3197-3206 (2006).

12 Lie, T. J., Pitta, T., Leadbetter, E. R., Godchaux, W., 3rd & Leadbetter, J. R. Sulfonates: novel electron acceptors in anaerobic respiration. *Arch Microbiol* **166**, 204-210 (1996).

13 Lie, T. J., Godchaux, W. & Leadbetter, E. R. Sulfonates as terminal electron acceptors for growth of sulfite-reducing bacteria (Desulfitobacterium spp.) and sulfate-reducing bacteria: effects of inhibitors of sulfidogenesis. *Appl Environ Microbiol* **65**, 4611-4617 (1999).

14 Laue, H., Denger, K. & Cook, A. M. Taurine reduction in anaerobic respiration of Bilophila wadsworthia RZATAU. *Appl Environ Microbiol* **63**, 2016-2021 (1997).

15 Cook, A. M. & Denger, K. Dissimilation of the C2 sulfonates. *Arch Microbiol* **179**, 1-6 (2002).
