# Supplementary material for: Radical-mediated C-S bond cleavage in C2 sulfonate degradation by anaerobic bacteria
Source: Nat Commun. 2019 Apr 8;10:1609. doi: 10.1038/s41467-019-09618-8 (PMC6453916; doi:10.1038/s41467-019-09618-8)
Supplement: Supplementary file 3 — Description of Additional Supplementary Files [file 41467_2019_9618_MOESM3_ESM.pdf]

Description of Additional Supplementary Files

File Name: Supplementary Data 1
Description: Source data for Supplementary Fig. 2

File Name: Supplementary Data 2
Description: *D. piger* protein mass spec source data for Fig. 4a

File Name: Supplementary Data 3
Description: *B. wadsworthia* with isethionate mass spec source data for Fig. 4b

File Name: Supplementary Data 4
Description: *B. wadsworthia* with taurine upper band mass spec source data for Fig. 4b

File Name: Supplementary Data 5
Description: *B. wadsworthia* with taurine lower band mass spec source data for Fig. 4b

File Name: Supplementary Data 6
Description: Codon optimized genes
