# Supplementary material for: Radical-mediated C-S bond cleavage in C2 sulfonate degradation by anaerobic bacteria
Source: Nat Commun. 2019 Apr 8;10:1609. doi: 10.1038/s41467-019-09618-8 (PMC6453916; doi:10.1038/s41467-019-09618-8)
Supplement: Supplementary file 2 — Peer Review File [file 41467_2019_9618_MOESM2_ESM.pdf]

Editorial Note: This manuscript has been previously reviewed at another journal that is not operating a transparent peer review scheme. The manuscript was considered suitable for publication without further review at *Nature Communications*.
